# Effect of different acupuncture modalities on sex hormone levels in perimenopausal women: a network meta-analysis

**DOI:** 10.3389/fendo.2026.1880483

**Published:** 2026-07-24

**Authors:** Shuyue Pang, Zhongtian Wang, Lili Zhang, Qi Qin, Xiuxiang Zhang, Rui Ma, Yanping Wang

**Affiliations:** 1 College of Traditional Chinese Medicine, Changchun University of Chinese Medicine, Changchun Jilin, China; 2 Children's Diagnosis and Treatment Center, The Affiliated Hospital of Changchun University of Chinese Medicine, Changchun, Jilin, China; 3 Obstetrics and Gynecology Diagnosis and Treatment Center, The Affiliated Hospital of Changchun University of Chinese Medicine, Changchun, Jilin, China; 4 Research Center of Traditional Chinese Medicine, The Affiliated Hospital of Changchun University of Chinese Medicine, Changchun, Jilin, China

**Keywords:** acupuncture, meta-analysis, nmaneuroendocrine regulation, perimenopause, sex hormones

## Abstract

**Background:**

Acupuncture, a common intervention in traditional Chinese medicine (TCM), has been widely applied for regulating hormonal fluctuations in perimenopausal women. However, the efficacy of various acupuncture modalities is not comprehensively compared. This network meta-analysis (NMA) evaluated the effects of different acupuncture approaches on sex hormone levels, specifically estradiol (E2), follicle-stimulating hormone (FSH), and luteinizing hormone (LH), in perimenopausal women.

**Methods:**

PubMed, Embase, the Cochrane Library, Web of Science, CNKI, Wanfang, and the VIP Database were searched until March 17, 2026. Randomized controlled trials (RCTs) on acupuncture interventions for perimenopausal women were included. The risk of bias was assessed for included studies, and data were synthesized based on a Bayesian NMA framework.

**Results:**

36 studies on 1, 480 participants were included. Compared with control interventions, acupoint application (AA) and acupuncture therapy (AT) significantly reduced Kupperman scores. In terms of FSH reduction, AA, abdominal moxibustion (AM), and AT were superior to control. For LH, AA, AT, and AM demonstrated notable reductions compared with control. As for E2 elevation, AA, AT, and compression acupuncture (PN) achieved statistically significant improvements.

**Conclusions:**

AM, AT, and AA significantly improved Kupperman scores, with AM being the most effective. Catgut implantation acupoint (CA) and PN showed significant effects in reducing FSH levels, followed by AT and AM, whereas plum blossom needling exhibited the greatest efficacy in increasing E2 levels. Overall, acupuncture presents a promising non-pharmacological strategy for modulating hormonal balance and alleviating menopausal symptoms in perimenopausal women.

**Systematic review registration:**

https://www.crd.york.ac.uk/PROSPERO/, identifier CRD420251066613.

## 1 Introduction

Perimenopause is the transitional period surrounding the cessation of menstruation and is characterized by a gradual decline in ovarian function. It typically occurs between the ages of 45 and 55 (1) and is one of the most important periods of physiological and psychological transformation in a woman’s life (2). During this period, ovarian function progressively declines, and sex hormone levels fluctuate considerably, particularly estradiol (E2), resulting in endocrine alterations. These changes are often accompanied by elevated follicle-stimulating hormone (FSH) and luteinizing hormone (LH) levels (3, 4), leading to vasomotor symptoms (e.g., hot flashes and night sweats), sleep disturbances, mood changes, menstrual irregularities, and osteoporosis, all of which may adversely affect quality of life (5). With accelerated population aging, the number of women experiencing perimenopause continues to increase worldwide, making the management of perimenopausal symptoms an important public health concern (6). Hormone replacement therapy (HRT) remains one of the most effective treatments for perimenopausal symptoms and works primarily through the supplementation of exogenous estrogen (7). However, long-term use of HRT has been associated with an increased risk of adverse events, including breast cancer, endometrial cancer, and thromboembolic disease, which may limit its acceptance and long-term use among some women (8, 9). Therefore, interest in non-pharmacological approaches has increased in recent years. Among these approaches, acupuncture has been widely used for the management of perimenopausal symptoms because of its favorable safety profile and broad clinical applicability (10).

By stimulating meridians and acupoints, acupuncture harmonizes yin and yang and modulates the function of internal organs, thereby achieving systemic homeostasis. Acupuncture may significantly regulate the neuroendocrine-immune (NEI) network (11). Several studies (12, 13) have proved that acupuncture may alleviate perimenopausal symptoms, regulate sex hormone levels, and potentially slow the decline in ovarian function. In recent years, many acupuncture modalities have been developed and applied in clinical practice, including traditional body acupuncture, electroacupuncture (EA), auricular acupuncture, abdominal acupuncture, and fire acupuncture (14). They differ in stimulation intensity, therapeutic mechanisms, point selection, and treatment frequency, so they have different clinical efficacy (15). For example, EA combines conventional acupuncture with electrical stimulation and may enhance treatment effects, whereas auricular acupuncture targets specific points on the ear and is often considered convenient and well accepted by patients. Acupuncture combined with traditional Chinese medicine (TCM) has also been investigated as a potential integrative treatment strategy (16, 17). Although these acupuncture modalities have been applied in clinical practice, their comparative effectiveness in modulating sex hormone levels among perimenopausal women remains inconclusive.

Although several systematic reviews and meta-analyses have evaluated acupuncture for perimenopausal syndrome (18, 19), most have focused on symptom relief or individual acupuncture interventions. Few network meta-analyses (NMAs) have compared multiple acupuncture modalities simultaneously using sex hormone levels as the primary physiological outcomes. Therefore, an NMA was conducted to compare and rank different acupuncture modalities with respect to their effects on serum E2, FSH, and LH levels in perimenopausal women, with the aim of providing evidence to support clinical decision-making.

## 2 Methods

This study was designed and reported according to the Preferred Reporting Items for Systematic Reviews and Meta-Analyses, incorporating Network Meta-Analyses (PRISMA-NMA) guidelines. The systematic review described herein was accepted by the online PROSPERO International Prospective Register of Systematic Reviews (20) of the National Institute for Health Research (CRD420251066613).

### 2.1 Eligibility criteria

Studies were eligible if they met the following criteria (1): randomized controlled trials (RCTs) (2); women diagnosed with perimenopause according to recognized diagnostic criteria (3); interventions involving acupuncture alone or acupuncture combined with pharmacotherapy; (4) comparison groups including sham acupuncture, no treatment, conventional treatment, or alternative acupuncture modalities; and (5) reporting at least one of the following outcomes: E2, FSH, LH, or the Kupperman Index. Studies were also required to provide sufficient data for quantitative analysis, including sample size, mean values, and standard deviations.

Studies were excluded if they: (1) were non-randomized studies; (2) evaluated acupuncture combined with other TCM interventions, such as Tuina or Gua Sha; (3) included women with non-natural menopause caused by oophorectomy or pituitary disorders; (4) enrolled participants with severe endocrine disorders; (5) did not report hormone-related outcomes; or (6) lacked extractable data.

### 2.2 Literature retrieval

A comprehensive systematic search was conducted across both English and Chinese databases, including PubMed, Embase, Cochrane Library, Web of Science, CNKI, China Biomedical Literature Database (CBM), Wanfang, and VIP Database. The search covered all literature published from the inception of each database through March 17, 2026. To ensure search comprehensiveness, both subject headings and free-text terms were employed. Search terms included “Acupuncture, ” “EA, ” “Auriculotherapy, ” “Auricular Acupuncture, ” “Scalp Acupuncture (SA), ” “Warm Acupuncture (WA), ” and “Perimenopause.” Search strategies were adapted to the specific syntax of each database. Moreover, the reference lists of relevant systematic reviews and grey literature sources (e.g., dissertations, conference abstracts) were manually screened to identify potentially eligible studies. Detailed search strategies are provided in **Supplementary Table 1**.

### 2.3 Data screening and extraction

Two reviewers independently selected studies and extracted data. All retrieved records were imported into EndNote 20, and duplicate records were removed. Titles and abstracts were screened initially, followed by full-text assessment according to the predefined inclusion and exclusion criteria. Any disagreements were resolved through discussion with a third reviewer.

Data were extracted using a standardized form, including: (1) first author and publication year; (2) sample size and mean age; (3) intervention type, treatment frequency, and treatment duration; and (4) outcome measures. When data were incomplete or presented only in graphical form, attempts were made to contact the corresponding authors for additional information. Any discrepancies in data extraction were resolved through consultation with a third reviewer.

### 2.4 Quality assessment

#### 2.4.1 Risk of Bias Assessment

The risk of bias for each included study was independently rated by two reviewers via the Cochrane Collaboration’s Risk of Bias Tool (21). Disagreements were resolved through discussion with a third reviewer. The assessment included random sequence generation, allocation concealment, blinding of participants and personnel, blinding of outcome assessment, incomplete outcome data, selective reporting, and other potential sources of bias. Studies were classified as having a low, unclear, or high risk of bias based on the assessment results. Studies meeting all criteria were considered low risk, those meeting some but not all criteria were considered unclear risk, and those failing to meet most criteria were considered high risk.

#### 2.4.2 Assessment of certainty of evidence

The Confidence in Network Meta-Analysis (CINeMA) framework was used to assess the certainty of evidence for each comparison. Six domains were evaluated: within-study bias, reporting bias, indirectness, imprecision, heterogeneity, and incoherence. Comparisons were downgraded for within-study bias when most studies contributing direct evidence were judged as having “some concerns” or “high risk” of bias. Prediction intervals were considered when assessing heterogeneity. The certainty of evidence for each comparison was categorized as high, moderate, low, or very low.

### 2.5 Data analysis

Bayesian NMA was conducted using R 4.3.2. Random-effects models with non-informative prior distributions were used to synthesize evidence across studies. Pooled effect estimates and treatment ranking probabilities were estimated using Markov chain Monte Carlo (MCMC) methods (22). For dichotomous outcomes, odds ratios with corresponding 95% credible intervals (CrIs) were reported. The surface under the cumulative ranking curve (SUCRA) was calculated to estimate the probability that each intervention was among the most effective treatments. Network plots were generated using STATA 15.0. In these plots, nodes represent interventions and edges represent direct comparisons, with node size proportional to the number of participants. Ranking plots were generated using the ggplot2 package in R. Subgroup analyses were performed according to treatment duration (<30 min vs ≥30 min). Sensitivity analyses were conducted using a leave-one-out method.

## 3 Results

### 3.1 Literature screening results

**Figure 1:**
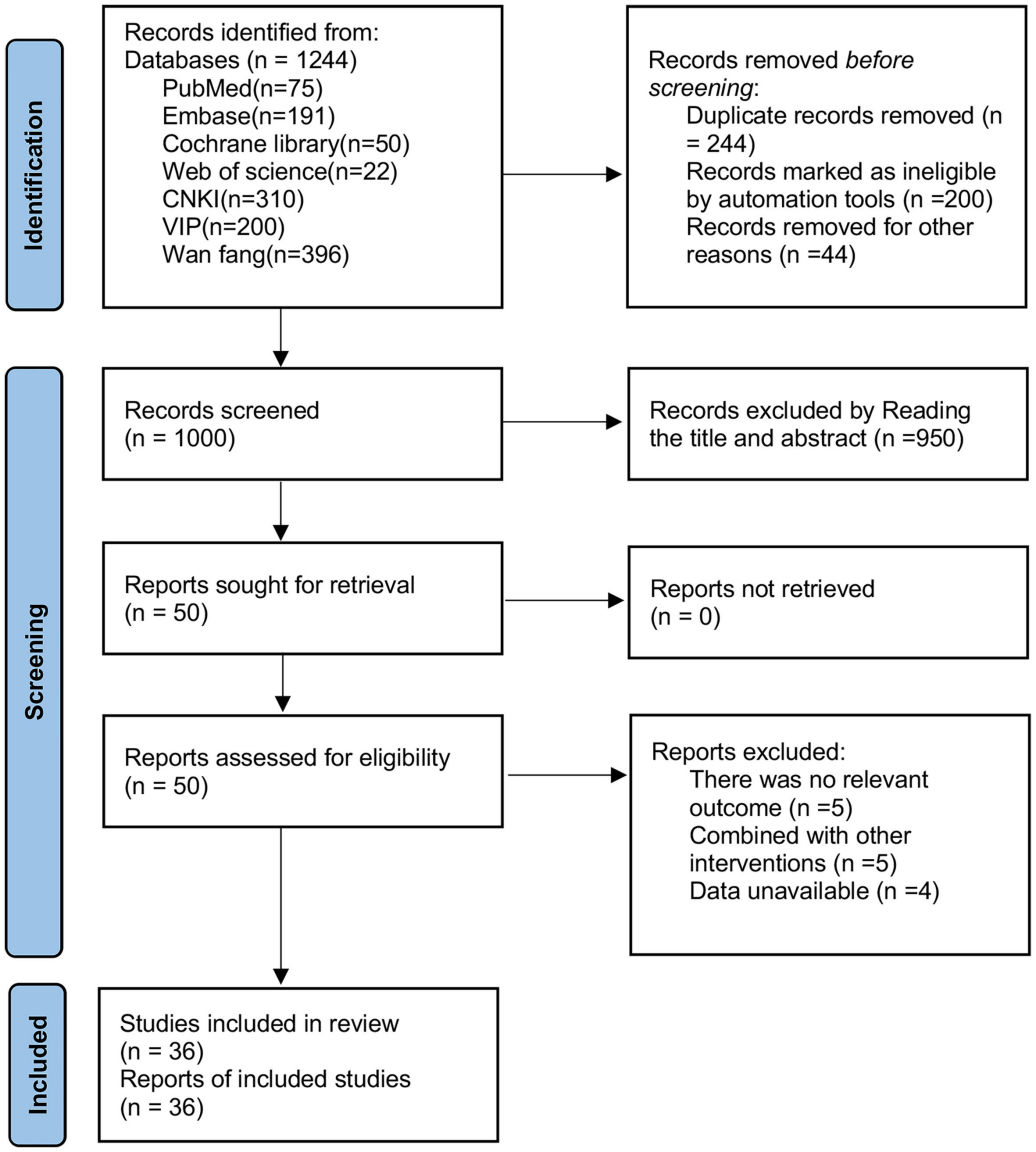
Literature search flow diagram.

As shown in **Figure 1**, the initial search yielded 1, 244 documents (PubMed (n=75), Embase (n=191), Cochrane Library (n=50), Web of Science (n=22), CNKI (n=310), VIP (n=200), Wanfang (n=396)). 244 duplicates were removed. Subsequently, 950 documents were excluded after title and abstract screening, and 14 were removed after their full texts were reviewed. Finally, 36 articles were analyzed (23–58).

### 3.2 Basic characteristics of the included literature

A total of 36 studies were included with a total sample size of around 1, 480 individuals. These studies involved 13 interventions: acupoint application (AA), EA, moxibustion (MB), hand acupuncture (HA), WA, catgut implantation acupoint (CA), abdominal moxibustion (AM), acupuncture therapy (AT), SA, and compression acupuncture (PN), AM_CM, AA_CM, EA_CM. Their mean age ranged from 45 to 53, with most being 48 and 51. The average age was 45-53, with most participants in the studies clustered between 48 and 51. Treatment frequency and duration varied across studies, ranging from once daily to three times weekly, with intervention periods extending from one month to 12 weeks. Detailed study characteristics are provided in **Table 1**.

**Table 1:** Table of basic characteristics.

Study	Year	Sample size	Mean age (years)	Intervention	Outcomes
QS Lan	2024	AA: 30 Control: 30	AA: 47.18 Control: 46.99	AA: Tianshu, Zhongwan, Guanyuan, Zusanli, Sanyinjiao, Twice/day, 30 minutes/each time	F2; F3; F4
J Liu	2016	EA: 14 Control: 15	EA: 48 Control: 48	EA: Guanyuan Tianshu Sanyinjiao. Twice/3 days, threetimes/week	F2; F3; F4
L sheng	2018	EA: 116 Control: 105	EA: 49.83 Control: 49.93	EA: Guanyuan, Zigong, Tianshu, Sanyingjiao, Hegu, Taichong, Baihui, Yintang, threetimes/week, 12 consecutive weeks	F2; F3; F4
FY Zhao	2023	AT:35 SA:35	RA:48.94 SA:48.80	AT: Yintang, Baihui, Guanyuan, Yinjiao, Neiguan, Taixi, Taichong, Sanyinjiao, Zigong, threetimes/week, 30 minutes/each time	F2; F3; F4
JH Zhou	2022	EA:108 Control:104	EA: 50 Control: 50	EA: BAIHUI, YINTANG, GUANYUAN, ZIGONG, TIANSHU, HEGU, TAICHONG, SANYINJIAO, three times/week	F2; F3; F4
CL Bao	2015	WA: 30 Control: 30	WA: 51 Control: 51	WA: Sanyinjiao, Taichong, Zusanli, Qi Hai, Guanyuan, Huiyuan, Neiguan, Tianshu, Baihui, Shenmen, Hegu. Zhao Hai Tai Xi, Shen Shu _ Ming Men, Three/week 30 minutes/each time	F2; F3; F4
W Q Bi	2020	AM: 40 MB: 39	AM: 47.88 MB: 48.23	AM: Origin: Yellow, mountain meat, Ligustrum lucidum, cow knee, lily, wheat winter Once/week 45 minutes/each time	F2; F3; F4
SS Cao	2016	MB: 47 Control: 47	MB: 47.3 Control: 48.1	MB: Sanyinjiao Guanyuan Shenyu, Fifteen times/month, 15 minutes/each time	F1;F2; F3; F4
HL Cao	2017	EA: 22 HA: 27	EA: 48 HA: 48	EA: Guanyuan, tianshu, Sanyinjiao, Three/week 30 minutes/each time	F2; F3; F4
R Cen	2018	CA: 30 EA: 30	CA: 49.34 EA: 48.85	CA: Zigong shen shu Mingmen ganshu xinshu, Once/week 1minutes/each time	F2; F3; F4
J X Che	2020	AT: 40 Control: 40	AT: 49.32 Control: 48.37	AT: ganshu shenshu zusanli sanyinjiao neiguan, Two months	F2; F3; F4
L Chen	2023	WA: 50 Control: 50 Control: 50	WA: 50.06 Control: 49.64 Control: 50.04	WA: Baihui Shenmen Sanyinjiao Xuanzhong, Once/day 30 minutes/each time	F1;F2; F3; F4
JH Gu	2022	AT: 45 Control: 45	AT: 49.83 Control: 49.21	AT: Baihui Shangxing Anmian sanyinjiao zusanli, Five/week 30 minutes/each time	F2; F3; F4
JR Yang	2017	SA: 81 Control: 81	SA: 48.17 Control: 49.45	SA: neiguan shenmen zusanli zhongwan, Fifteen/month, 30 minutes/each time	F2; F3; F4
RH Wei	2021	AM: 30 Control: 30	AM: 49.7 Control: 50.3	AM: guanyuan Zusanli, sanyinjiao, Twice/week, 30 minutes/each time	F1;F2; F3; F4
S Liu	2020	AA: 60 Control: 60	AA: 49.55 Control: 49.61	AA: sanyinjiao neiguan, Three four/hours	F2; F3; F4
YZWCai	2017	HA: 25 EA: 25	HA: 51 EA: 51	HA: guanyuan zigong tianshu sanyinjiao, Three/week 30 minutes/each time	F2; F3; F4
YM Xu	2017	WA: 30 Control: 30	WA: 49.53 Control: 50.16	WA: guanyuan tianshu zigong sanyinjiao, Once/month	F2; F3; F4
YR Wang	2018	AA: 56 AM: 57	AA: 51.9 AM: 51.9	AA: guanyuan shenshu ganshu zigong, One/day 30 minutes/each time	F1;F4
JL Lin	2024	EA: 38 Control: 38	EA: 53.73 Control: 53.86	EA: tianshu sanyinjiao guanyuan zigong, Three/week 30 minutes/each time	F2; F3; F4
Y Lu	2022	AM: 46 Control: 46	AM: 46.13 Control: 46.13	AM: baihui guanyuan zigong tianshu sanyinjiao, Five/week 30 minutes/each time	F2; F3
Y Ou	2024	AA: 48 Control: 48	AA: 49.90 Control: 49.64	AA: guanyuan zigong ganshu sanyinjiao, One/day three hours/each time	F2; F3; F4
YL Lou	2023	AM: 35 Control: 35	AM: 45.97 Control: 46.07	AM: yongquan shuenshu sanyinjiao guanyuan, One/day 20 minutes/each time	F2; F3; F4
XL Shi	2011	EA: 40 Control: 40	EA: 51.15 Control: 52.3	EA: guanyuan sanyinjiao baihui fengchi neiguan, Three/week 30 minutes/each time	F1;F2;
HH Liu	2021	AA: 30 Control: 30	AA: 51.9 Control: 52.2	AA: neiguan waiguan sanyinjiao, Once/day three hours/each time	F1;F2; F3; F4
R Wang	2020	AM: 30 Control: 30	AM: 49.5 Control: 50.2	AM: shenshu mingmen guanyuan, Once/day 10 minutes/each time	F1;F2; F3; F4
XL Lv	2017	AM: 38 Control: 38	AM: 48.6 Control: 47.7	AM: baihui xinshu shenshu guanyuan shemen, Once/day 30 minutes/each time	F2; F3; F4
P Zhou	2014	AM: 39 Control: 39	AM: 47.62 Control: 48.41	AM: sanyinjiao baihui shenshu zusanli, Fifteen/month	F2; F3
QF Tan	2020	EA: 50 Control: 50	EA: 47.32 Control: 48.06	EA: guanyuan sanyinjiao ganshu, Once/day 30 minutes/each time	F2; F3; F4
YP Zhou	2021	AM: 40 Control: 40	AM: 49.8 Control: 48.94	AM: neiguan baihui shenmen sanyinjiao guanyuan zusanli, 10 minutes/each time	F2; F3; F4
ZY Qing	2007	EA: 90 Control: 85	EA: 48 Control: 45	EA: sanyinjiao, Three/week 30 minutes/each time	F2; F3; F4
LY Lin	2021	WA: 38 Control: 39	WA: 49.31 Control: 48.12	WA: shenshu ganshu sanyinjiao yangling, Once/10day	F2; F3; F4
HL Huang	2017	AM_CM: 30 Control: 30	AM_CM: 50 Control: 50	AM: Baihui Sishen guanyuan qihai Sanyinjiao, Three/week 30 minutes/each time	F2; F3; F4
Y Yang	2020	PN: 50 Control: 50	PN: 40.22 Control: 40.56	PN: Shemen, 30 minutes/each time	F2; F3
XJ Sun	2023	AT: 53 Control: 53	AT: 51.62 Control: 51.66	AT: baihui, yintang, fengchi, zhaohai, shenmen, sanyinjiao, xinshu, anmian, neiguan 30 minutes/each time, Three/week	F2; F3; F4
Yx Song	2018	AT: 46 Control: 46	AT: 49.51 Control: 49.33	AT: qihai, ganshu, shenshu, shenmen, sanyinjiao, taixi, yingu, zhaohai 30 minutes/each time, Three/week	F1;F2; F3; F4

AA, Acupoint application; EA, Electro acupuncture; MB, Moxibustion; HA, Hand_ acupuncture; WA, warm acupuncture; CA, catgut implantation acupoint; AM, Abdominal moxibustion; AT, Acupuncture therapy; SA, scalp acupuncture; PN, Press needle. CM, Chinese medicine. F1, Kupperman; F2, FSH; F3, LH; F4, E2.

### 3.3 Risk of bias in results

**Figure 2:**
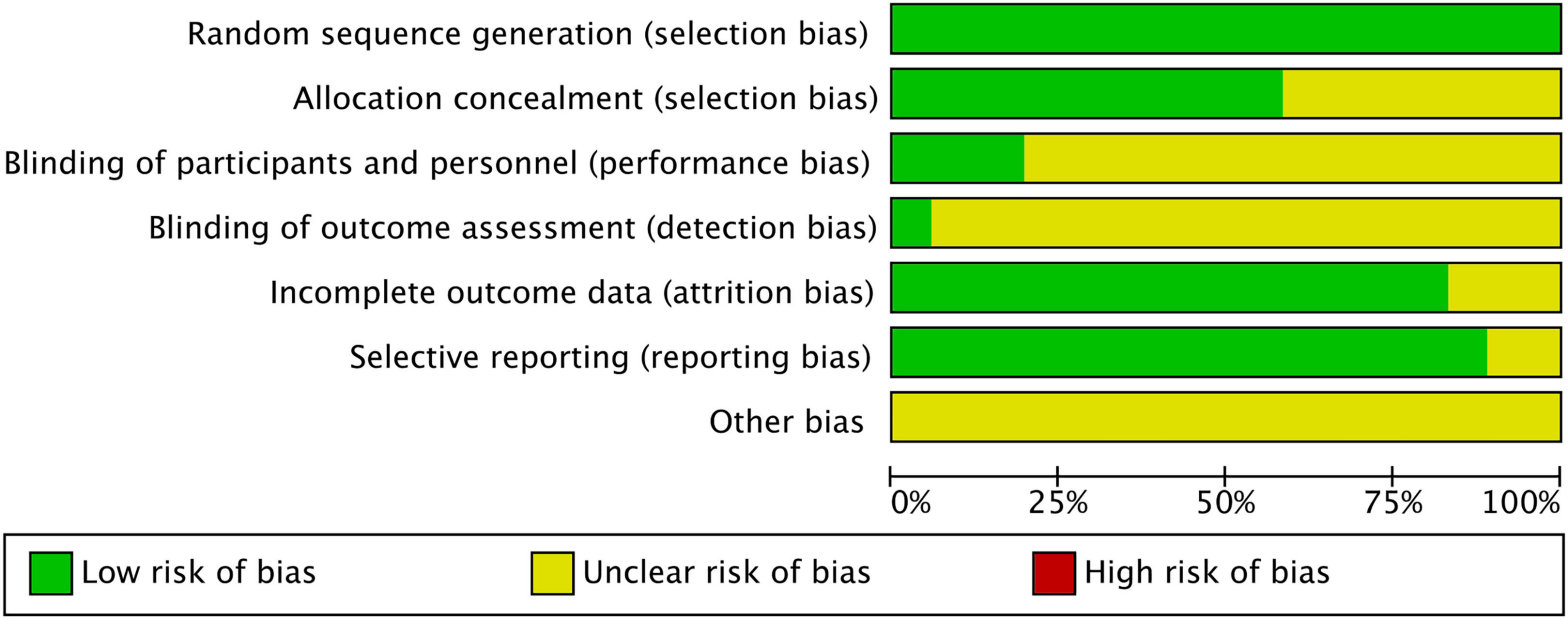
Risk of bias graph.

**Figure 3:**
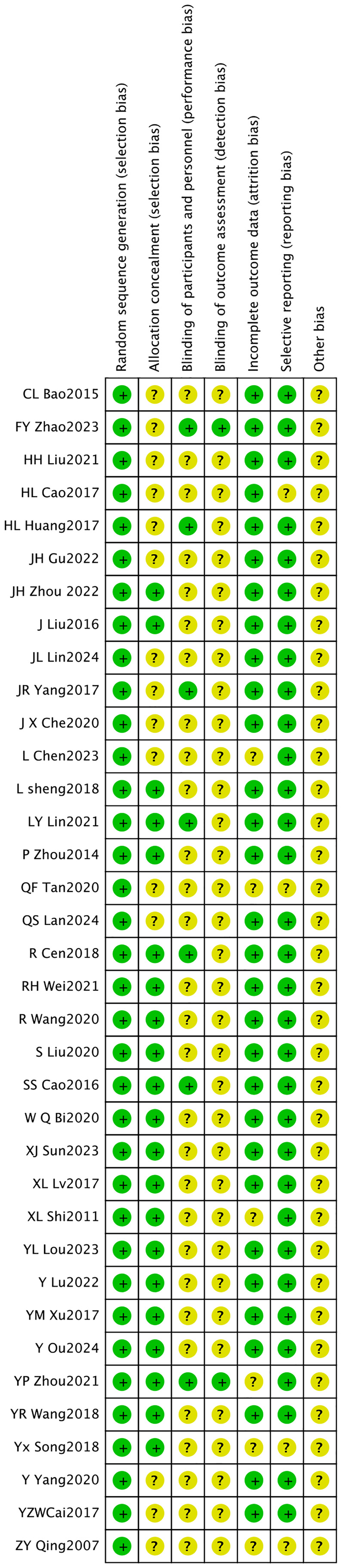
Risk of bias summary.

All included studies clearly described their methods of random sequence generation and were therefore judged to have a low risk of selection bias for this domain. For the remaining domains, including allocation concealment, blinding of participants and personnel, blinding of outcome assessment, incomplete outcome data, selective reporting, and other potential sources of bias, the risk of bias varied across studies. Detailed risk-of-bias assessments and overall study quality ratings are presented in **Figures 2**, **3**.

### 3.4 Certainty of the evidence

**Supplementary Figures 6**–**9** show the strength of evidence for each network estimate. Overall, the level of certainty of evidence was very low.

### 3.5 Meta-analysis results

#### 3.5.1 Kupperman score

**Figure 4:**
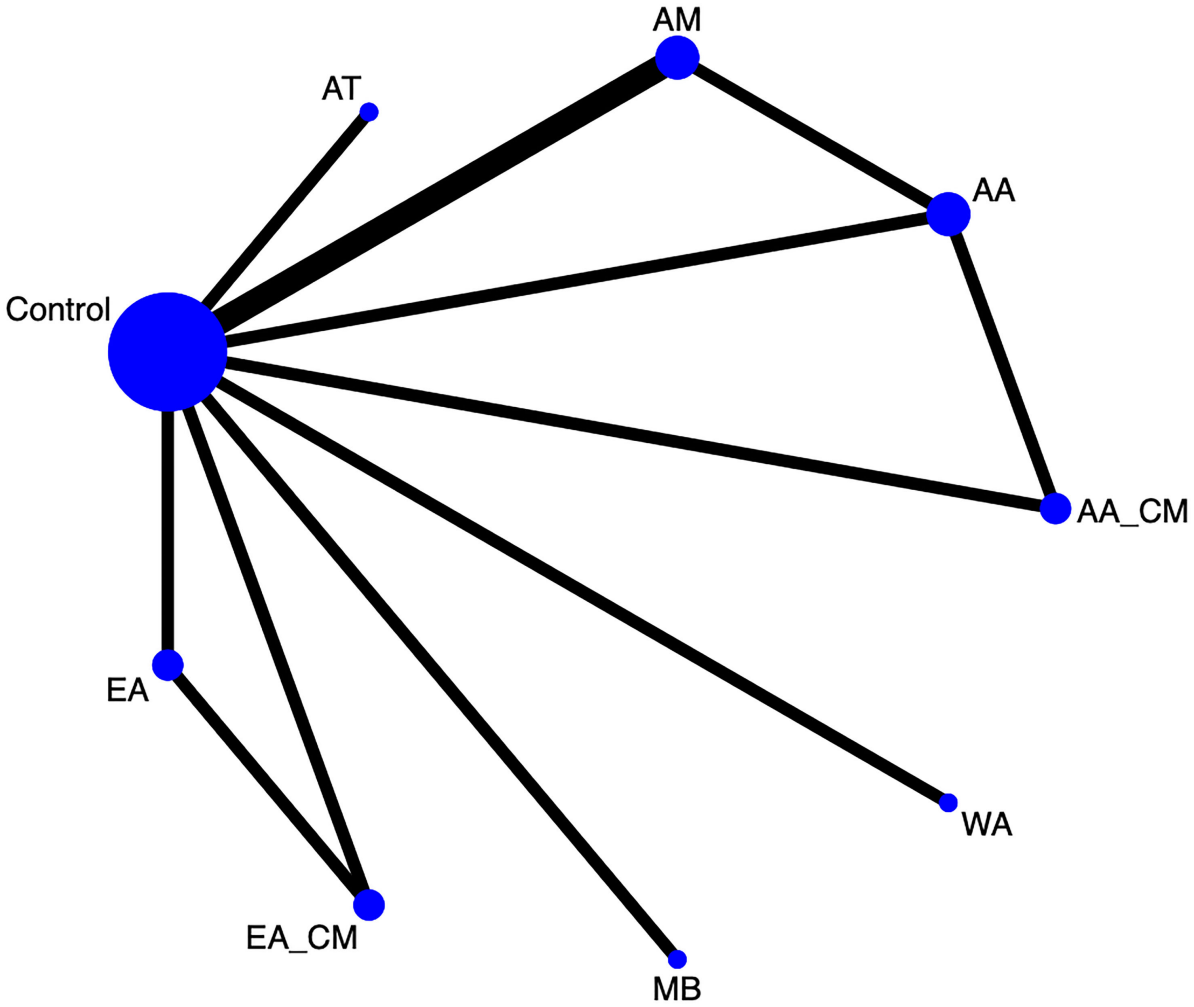
Network plot for Kupperman score.

**Figure 5:**
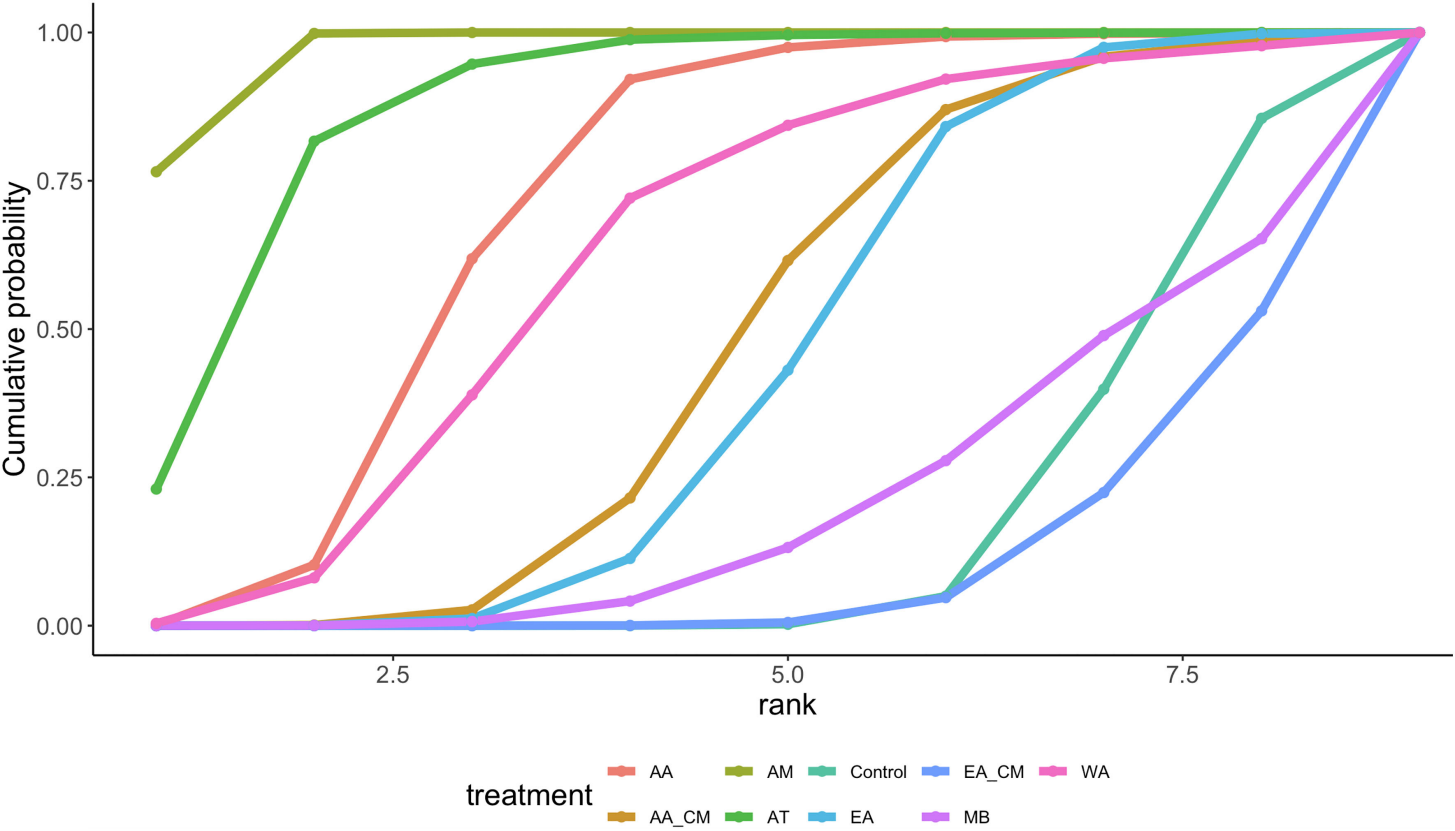
Cumulative ranking probability plot for Kupperman score.

Eight studies reported Kupperman scores, and the corresponding network diagram (**Figure 4**) formed a closed loop. Therefore, a local inconsistency test was conducted, which indicated no statistically significant differences among direct comparisons, indirect comparisons, and network comparisons for AM versus AA, Control versus AA, and Control versus AM (**Supplementary Figure 1**). The league table (**Supplementary Table 2**) demonstrated that, compared with Control, AA [MD=-2.38, 95% CI (-4.00, -0.74)], AM [MD=-5.04, 95% CI (-5.58, -4.49)], and AT [MD=-4.09, 95% CI (-6.56, -1.64)] were effective in reducing the Kupperman score. Furthermore, AM exhibited superior efficacy compared with EA [MD=-3.29, 95% CI (-5.91, -0.67)] and MB [MD=-4.06, 95% CI (-7.01, -1.13)]. The SUCRA curve (**Table 2**; **Figure 5**) indicated the following probability rankings: AM (97%) > AT (87.2%) > AA (70.1%).

**Table 2:** SUCRA rankings.

Treatment	Kupperman (%)	FSH(%)	LH(%)	E2(%)
AA	70.1	48.2	58.2	46.3
AA_CM	45.9	74.5	73.8	NR
AM	97.0	61.3	30.6	41.7
AM_CM	NR	NR	74.8	81.5
AT	87.2	66.4	63.2	66.6
CA	NR	89.5	97.7	66.7
Control	16.3	20.4	0.72	16.7
EA	42.1	38.9	13.6	9.49
HA	NR	32.1	67.5	4.30
MB	20.0	20.1	34.7	56.2
PN	NR	87.0	NR	99.3
SA	NR	4.12	33.7	83.2
EA_CM	10.1	NR	NR	NR
WA	61.2	63.5	51.3	28.1

#### 3.5.2 FSH

**Figure 6:**
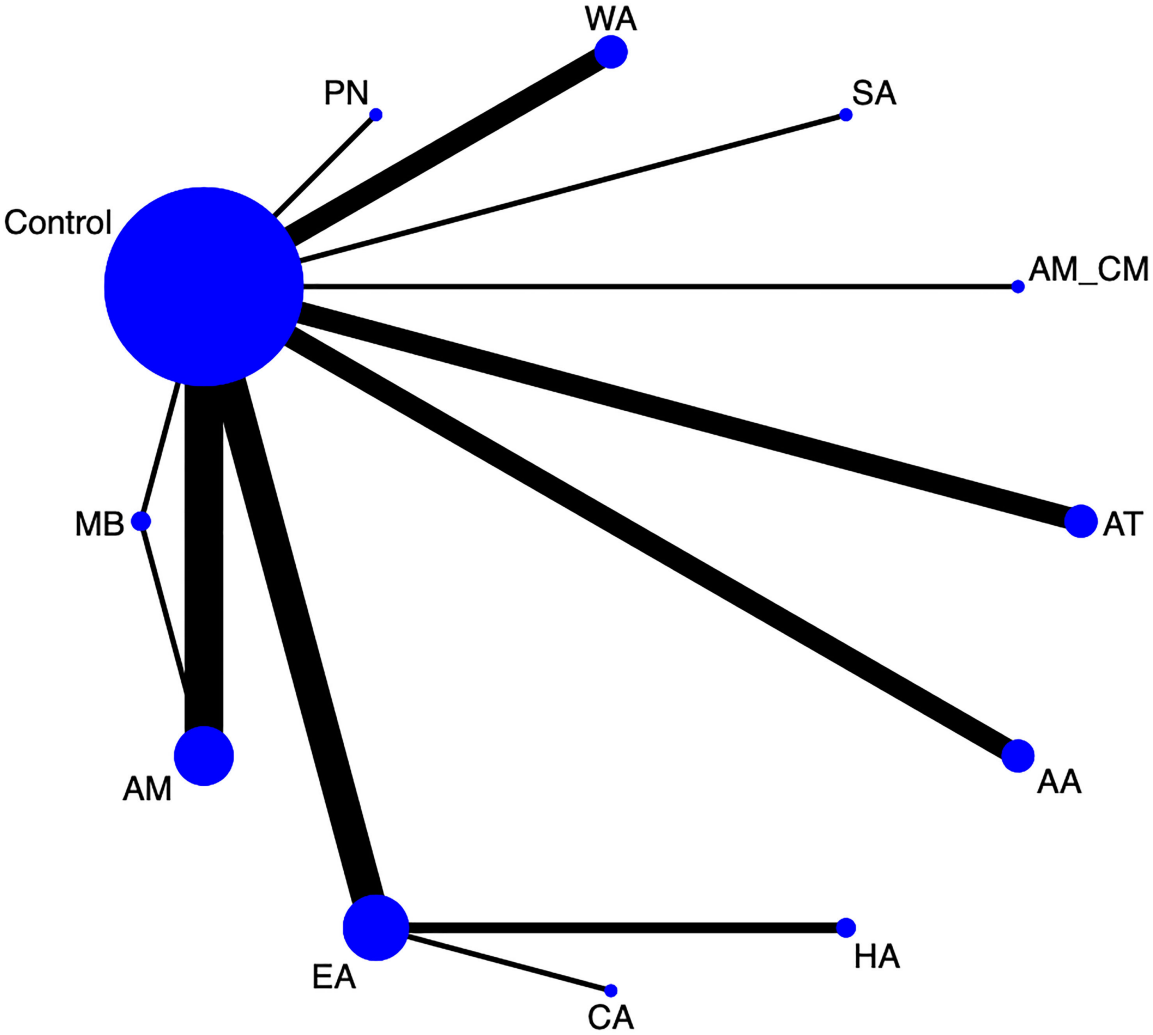
Network plot for FSH.

**Figure 7:**
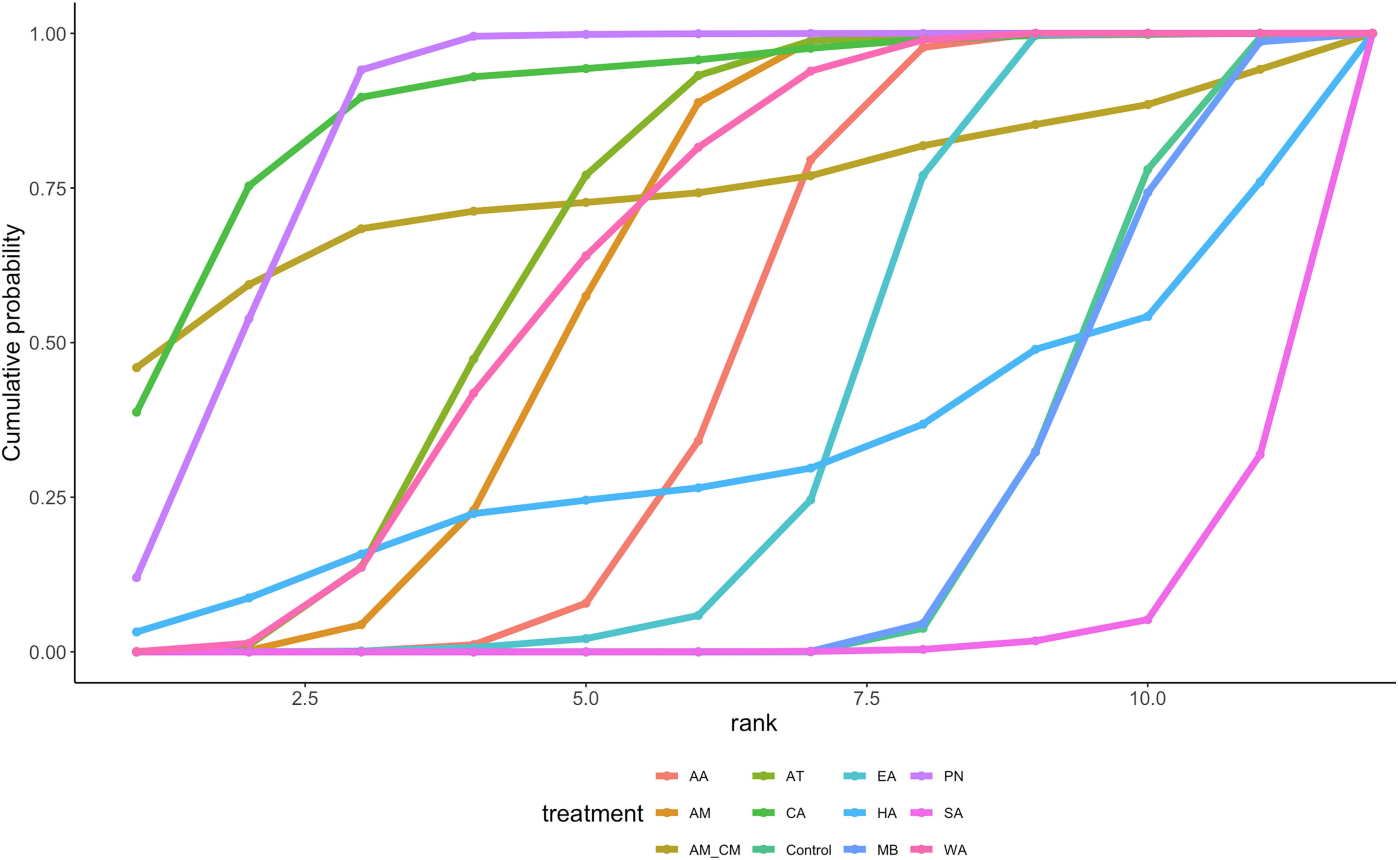
Cumulative ranking probability plot for FSH.

34 studies reported FSH levels, and the corresponding network (**Figure 6**) formed a closed loop. Therefore, a local inconsistency test was performed, and the results (**Supplementary Figure 2**) showed no significant differences among direct, indirect, and network comparisons for Control versus AM, MB versus AM, and MB versus Control. According to the league table (**Supplementary Table 3**), AA [MD=-3.73, 95% CI (-4.24, -3.23)], AM [MD=-4.34, 95% CI (-4.90, -3.79)], and AT [MD=-4.64, 95% CI (-5.61, -3.68)] significantly reduced FSH levels compared with Control. Moreover, AT was more effective than MB [MD=-4.67, 95% CI (-6.37, -2.98)]. The SUCRA curve (**Table 2** and **Figure 7**) showed the following rankings: CA (89.5%) > PN (87%) > AA_CM (74.5%).

#### 3.5.3 LH

**Figure 8:**
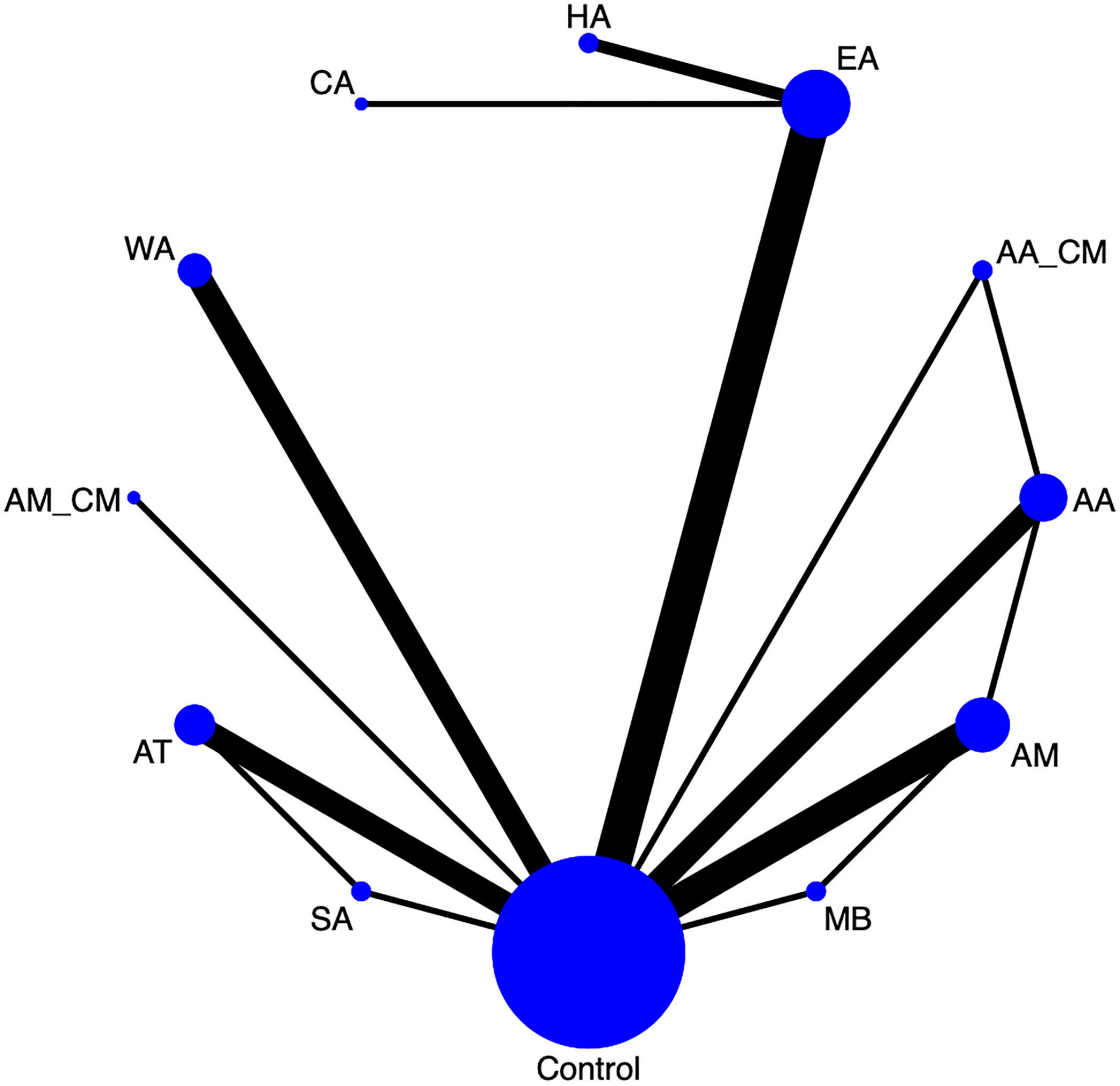
Network plot for LH.

**Figure 9:**
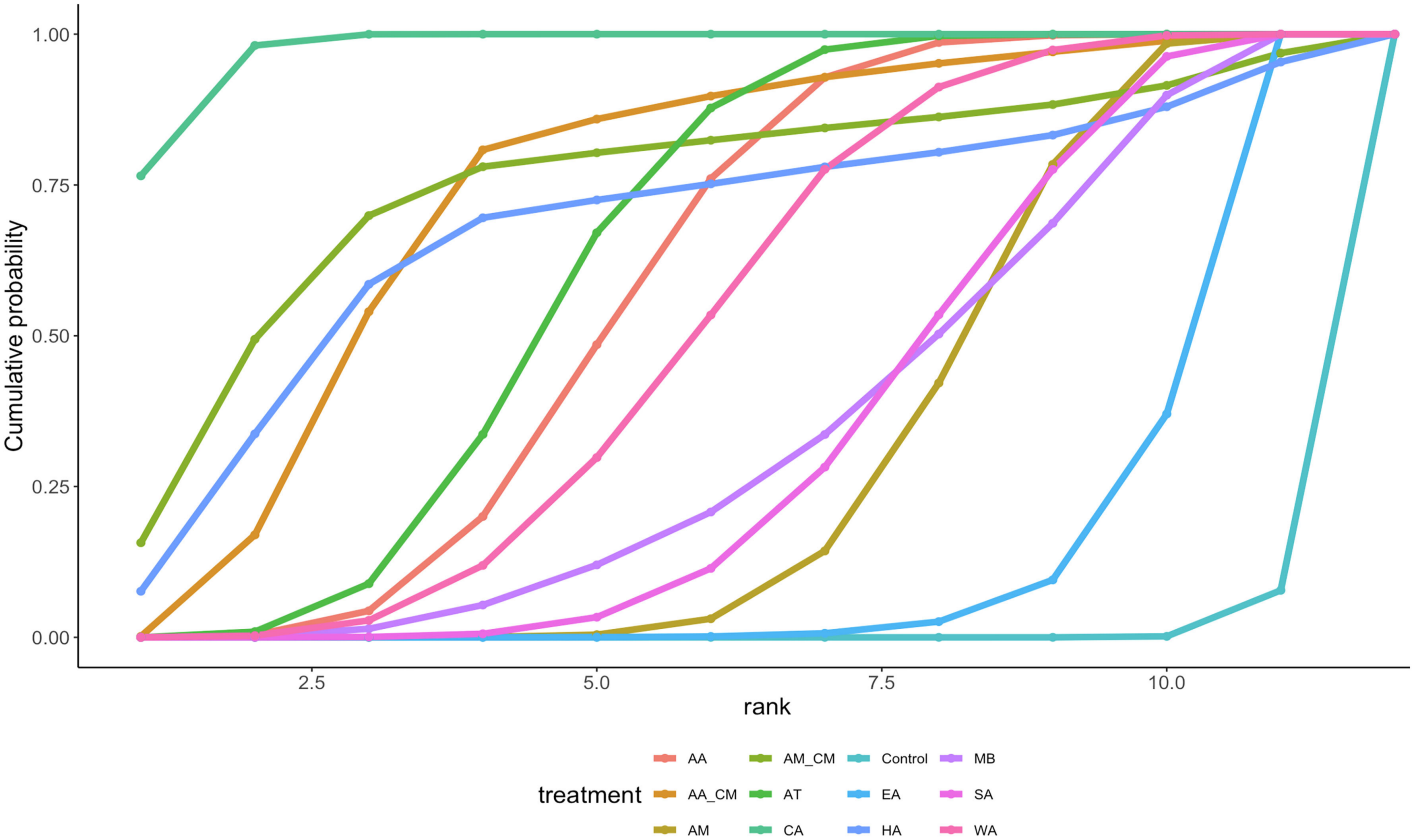
Cumulative ranking probability plot for LH.

32 studies reported LH levels, and the network (**Figure 8**) formed a closed loop. A local inconsistency test was therefore applied, and the findings (**Supplementary Figure 3**) revealed no significant differences in direct, indirect, and network comparisons for the following pairs: AM versus AA, Control versus AA, Control versus AM, MB versus AM, Control versus AT, SA versus AT, MB versus Control, and SA versus Control. The league table (**Supplementary Table 4**) indicated that AA [MD=-4.09, 95% CI (-4.86, -3.32)], AT [MD=-4.28, 95% CI (-5.09, -3.47)], and AM [MD=-3.11, 95% CI (-3.68, -2.53)] significantly decreased LH levels when compared with Control. Furthermore, CA demonstrated superior efficacy over EA [MD=-9.49, 95% CI (-11.82, -7.15)], MB [MD=-8.41, 95% CI (-11.46, -5.37)], and SA [MD=-8.38, 95% CI (-11.13, -5.62)]. The SUCRA curve (**Table 2**; **Figure 9**) revealed the following rankings: CA (97.7%) > AM_CM (74.8%) > AA_CM (73.8%).

#### 3.5.4 E2

**Figure 10:**
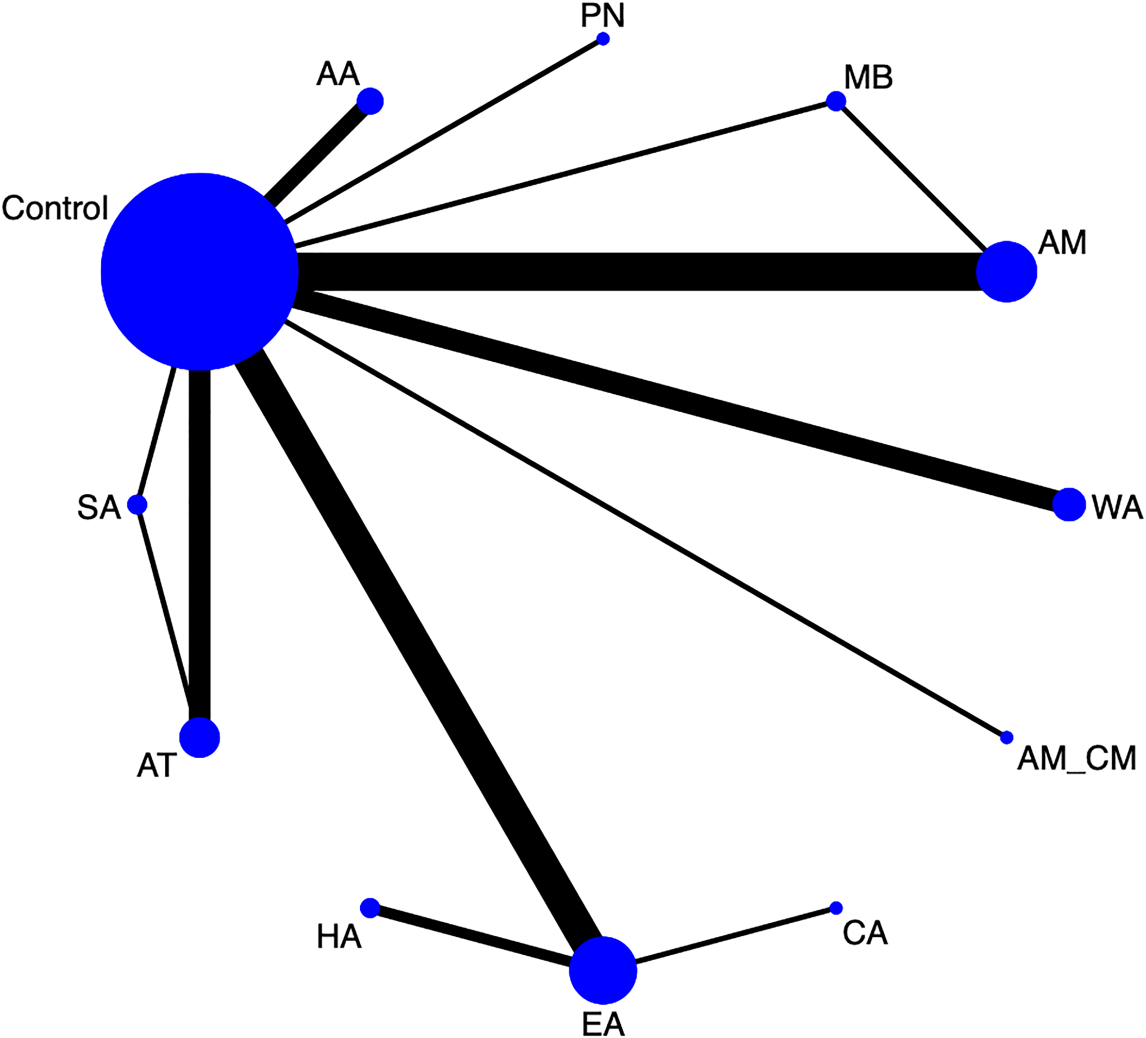
Network plot for E2.

**Figure 11:**
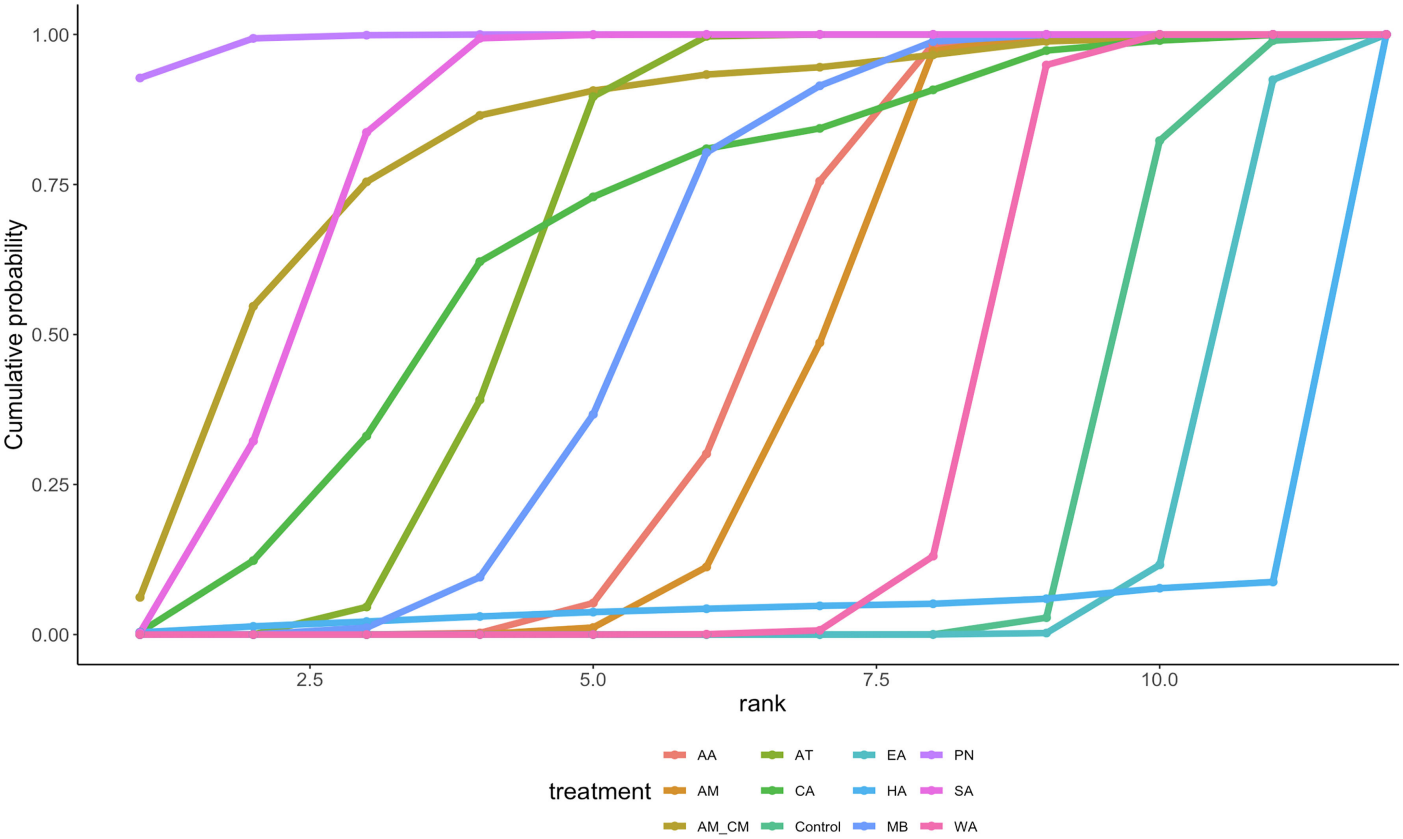
Cumulative ranking probability plot for E2.

32 studies reported E2 levels, and the network (**Figure 10**) formed a closed loop. A local inconsistency test was therefore conducted, and the results (**Supplementary Figure 4**) suggested no statistically significant differences among direct, indirect, and network comparisons for SA versus Control. The league table (**Supplementary Table 5**) demonstrated that AA [MD = 5.15, 95% CI (3.94, 6.36)], AM [MD = 4.8, 95% CI (4.28, 5.31)], AT [MD = 7.81, 95% CI (7.00, 8.63)], and PN [MD=-26.9 (-37.29, -16.58)] significantly increased E2 levels compared with Control. PN was also more effective than SA [MD = 13.94, 95% CI (2.94, 24.95)] and WA [MD = 23.86, 95% CI (13.46, 34.34)]. The SUCRA curve (**Table 2**; **Figure 11**) showed the following probability rankings: PN (99.9%) > SA (83.2%) > AM_CM (81.5%).

### 3.6 Publication bias

Publication bias was assessed using funnel plots. Visual inspection suggested the possibility of publication bias for E2, FSH, LH, and Kupperman scores (**Supplementary Figures 5**–**8**).

### 3.7 Subgroup analysis

Subgroup analyses were performed according to treatment duration (<30 min vs ≥30 min). The results for each outcome are presented in **Supplementary Figures 9**–**12**.

Overall, treatment duration did not appear to substantially influence the direction of treatment effects or the ranking of interventions across outcomes.

### 3.8 Sensitivity analysis

Sensitivity analyses were performed using a leave-one-out approach by sequentially removing each intervention node from the network. Changes in SUCRA values and treatment rankings were then evaluated. The results (**Supplementary Figures 13**–**15**) showed that the highest-ranked interventions remained largely unchanged across Kupperman score, FSH, LH, and E2 outcomes. Removal of individual intervention nodes resulted in only minor changes in SUCRA values for the remaining treatments, and the overall ranking order was generally preserved. Moreover, the evidence networks for each outcome (**Supplementary Figure 16**) demonstrated an adequate distribution of studies and sample sizes, together with a sufficient number of direct comparisons. These findings reflect the robustness of the NMA results.

## 4 Discussion

### 4.1 Main findings

In this study, the effects of different acupuncture interventions on sex hormone levels in perimenopausal women were evaluated using network meta-analysis. The results suggested that AM, AT, and PN were associated with improvements in perimenopausal symptoms, reductions in FSH and LH levels, and increases in E2 levels. Among the evaluated interventions, AM ranked highest for reducing Kupperman scores and FSH levels. EA and MB also showed beneficial effects, although their estimated effects were generally smaller than those of AM and PN. However, the number of studies evaluating interventions such as CA and PN was limited. Therefore, their ranking results require interpretation with caution.

The Kupperman score was used to assess the improvement of common symptoms in perimenopausal women, like hot flashes, insomnia, and mood swings (59). The analysis indicated that AM was the most effective modality in reducing Kupperman scores, followed by AT and acupuncture combined with TCM (AA). AM exerts its therapeutic effect by stimulating specific acupoints to enhance the flow of qi and blood and to restore organ function, demonstrating particular efficacy in regulating body temperature and alleviating vasomotor symptoms such as hot flashes and night sweats. Compared with other treatments such as EA and MB, AM exhibited notably superior outcomes, likely attributable to its robust local stimulation and systemic regulatory effects. Although AT and MB also demonstrated beneficial effects on perimenopausal symptoms, their efficacy was slightly inferior to that of AM (19). With respect to FSH and LH, AM, AT, and MB were associated with reductions in both hormone levels. For FSH, SUCRA rankings suggested that CA and PN had the highest probability of being among the most effective interventions, followed by AA_CM and AT. For LH, CA ranked highest. Elevated FSH and LH levels are commonly regarded as indicators of declining ovarian function during perimenopause (60). Acupuncture is postulated to modulate this imbalance by regulating the hypothalamic-pituitary-ovarian (HPO) axis. The superior efficacy of AM may be associated with its warming stimulation of the abdominal region and its tonifying effects on the spleen and stomach, promoting qi and blood circulation and contributing to endocrine equilibrium (61). Moreover, AT and MB can also effectively regulate FSH and LH and relieve perimenopausal symptoms by stimulating specific acupoints. The efficacy of methods such as EA and MB also performed better in this regard, but was not as significant as that of AT. E2 modulation is important in the treatment of perimenopausal women, as a decrease in E2 causes many perimenopausal symptoms (62). AM, AT, and PN significantly increased E2 levels, with PN showing the most significant effect. As a deep stimulation technique, PN increases estrogen levels by promoting local circulation of qi and blood and stimulating endocrine gland secretion. Notably, PN yielded greater improvements in E2 levels than other interventions such as WA and SA, likely because it has better stimulation and regulatory capacity (63). MB and AT also performed well in improving E2, especially in the comprehensive treatment, and the combination of acupuncture and medicine (AA_CM, EA_CM) can improve the efficacy of the treatment (19).

### 4.2 Mechanisms

First, acupuncture may influence sex hormone secretion through modulation of the HPO axis. Gonadotropin-releasing hormone (GnRH) released by the hypothalamus stimulates the secretion of FSH and LH from the pituitary gland, which in turn regulate ovarian follicular development and sex hormone production (64). During perimenopause, declining ovarian function and fluctuating hormone levels may disrupt this regulatory pathway. Acupuncture may affect hypothalamic and pituitary activity and thereby contribute to hormonal regulation. Acupuncture may also improve neuropsychological symptoms such as mood changes and sleep disturbances through effects on neurotransmitter systems (65). It may influence the release of neurotransmitters, including serotonin (5-HT) and gamma-aminobutyric acid (GABA), which are involved in the regulation of anxiety, depression, and sleep (66). Specifically, acupuncture regulates the psychological state by stimulating specific acupoints, activating the nervous system, and enhancing nerve conduction. Moreover, acupuncture further exerts its regulatory effects by improving blood circulation and immune function. By stimulating local and distant points, acupuncture improves qi and blood circulation and improves microcirculation throughout the body, thereby improving the body’s immunity and enhancing self-repairing ability (10). This mechanism is important in alleviating perimenopausal symptoms, especially in alleviating symptoms of thermoregulatory dysfunction like hot flashes and sweating.

### 4.3 Clinical significance

The findings suggest that acupuncture interventions may have potential value in regulating sex hormone levels and improving perimenopausal symptoms. Interventions such as AM, AT, and PN were associated with improvements in vasomotor and neuropsychological symptoms and with favorable changes in FSH, LH, and E2 levels. These findings support the potential role of acupuncture as a non-pharmacological option for women seeking alternatives to long-term hormone replacement therapy. The flexibility of acupuncture treatment may also facilitate its integration into individualized management strategies during the menopausal transition.

### 4.4 Strengths and limitations

This study systematically evaluated the effects of different acupuncture modalities on hormonal regulation and symptom improvement in perimenopausal women through an NMA. Its greatest strength lies in its ability to synthesize findings from multiple independent studies. By employing NMA, it comprehensively compared treatment options and, even in the absence of head-to-head trials, ranked different interventions based on indirect evidence, offering more scientifically grounded guidance for clinical decision-making. In addition, acupuncture, as a non-pharmacological therapy, carries no risk of side effects or dependence, making it a safe and valuable alternative, particularly for women who prefer to avoid hormone replacement therapy. Its clinical relevance in this context is substantial. Furthermore, this study sheds light on the multiple mechanisms through which acupuncture alleviates perimenopausal symptoms, especially involving the regulation of the neuro-endocrine-immune network, thereby strengthening its theoretical foundation.

Although some meaningful conclusions were drawn through systematic literature screening and NMA, there are some limitations.

First, heterogeneity was present across the included studies with respect to intervention protocols, sample size, and follow-up duration, which may have affected the comparability of results. Although sensitivity and subgroup analyses were performed, some findings remained dependent on network structure and evidence availability. The leave-one-out analysis showed that removal of AA or AM resulted in SUCRA changes exceeding 20 percentage points and altered the highest-ranked intervention, suggesting that some rankings were influenced by a limited number of bridging studies. In addition, SUCRA values should be interpreted cautiously because they reflect ranking probabilities rather than absolute treatment effects. Their stability may be influenced by sample size, network structure, and evidence consistency. Interventions such as CA and PN were evaluated in relatively few studies, and their high rankings require further confirmation. Confidence intervals in subgroup analyses were generally wide, with several crossing the null value, indicating limited precision. Although the rankings for E2, FSH, and LH were relatively stable, some variation in effect estimates across subgroups remained, suggesting that heterogeneity was not fully explained by the subgroup analyses. Therefore, the ranking results should be regarded as exploratory and interpreted in conjunction with evidence quality and network connectivity.

Second, some studies did not report detailed hormone data or reported outcomes in formats that limited data extraction, which may have affected the precision and completeness of the analyses.

Third, although this study focused on acupuncture interventions, treatment responses may vary across individuals. In addition, most included studies were conducted in China, which may limit the generalizability of the findings to other populations. Future studies should evaluate treatment effects in more diverse populations and examine long-term outcomes.

Fourth, future research should prioritize large-scale, multicenter, rigorously conducted RCTs and include longer follow-up periods and more diverse study populations.

## 5 Conclusion

In this study, the effects of different acupuncture treatments in relieving menopausal symptoms and hormone level regulation were evaluated through an NMA. AM, AT, and AA had significant improvement on Kupperman scores, with AM having the most significant effect. For the regulation of FSH levels, CA and PN showed the highest SUCRA rankings, suggesting greater potential for reducing FSH. For LH levels, CA was superior to other interventions in reducing LH. For E2 levels, AA, AM, and PN all demonstrated efficacy in increasing E2, with PN showing the most pronounced effect. Overall, acupuncture-based interventions appear to have promising potential in regulating hormone levels and alleviating menopausal symptoms, particularly in increasing E2 while reducing FSH and LH.

## Data Availability

The original contributions presented in the study are included in the article/**Supplementary Material**. Further inquiries can be directed to the corresponding author.
