# Supplementary material for: Effect of different acupuncture modalities on sex hormone levels in perimenopausal women: a network meta-analysis
Source: Front Endocrinol (Lausanne). 2026 Jul 24;17:1880483. doi: 10.3389/fendo.2026.1880483 (PMC13447166; doi:10.3389/fendo.2026.1880483)
Supplement: Supplementary file 1 [file Table1.docx]

**Table S1 S**earch strategies

(("Acupuncture"[Mesh]) OR ((((((((((((((((((((((((((((((Acupuncture[Title/Abstract]) OR (Pharmacopuncture[Title/Abstract])) OR (Acupuncture Therapy[Title/Abstract])) OR (Acupuncture Treatment[Title/Abstract])) OR (Acupuncture Treatments[Title/Abstract])) OR (Treatment, Acupuncture[Title/Abstract])) OR (Therapy, Acupuncture[Title/Abstract])) OR (Pharmacoacupuncture Treatment[Title/Abstract])) OR (Treatment, Pharmacoacupuncture[Title/Abstract])) OR (Pharmacoacupuncture Therapy[Title/Abstract])) OR (Therapy, Pharmacoacupuncture[Title/Abstract])) OR (Acupotomy[Title/Abstract])) OR (Acupotomies[Title/Abstract])) OR (Acupuncture, Ear[Title/Abstract])) OR (Acupunctures, Ear[Title/Abstract])) OR (Ear Acupunctures[Title/Abstract])) OR (Acupuncture, Auricular[Title/Abstract])) OR (Acupunctures, Auricular[Title/Abstract])) OR (Auricular Acupunctures[Title/Abstract])) OR (Auricular Acupuncture[Title/Abstract])) OR (Ear Acupuncture[Title/Abstract])) OR (Acupuncture Points[Title/Abstract])) OR (Acupuncture Point[Title/Abstract])) OR (Point, Acupuncture[Title/Abstract])) OR (Points, Acupuncture[Title/Abstract])) OR (Acupoints[Title/Abstract])) OR (Acupoint[Title/Abstract])) OR (Electroacupuncture[Title/Abstract])) OR (Moxibustion[Title/Abstract])) OR (Moxabustion[Title/Abstract]))) AND (("Perimenopause"[Mesh]) OR (Perimenopause[Title/Abstract]))

**Table S2** Kupperman score League table

| MD 95%CI | | | | | | | | |
| --- | --- | --- | --- | --- | --- | --- | --- | --- |
| AA |  |  |  |  |  |  |  |  |
| -1.37 (-3.13, 0.43) | AA_CM |  |  |  |  |  |  |  |
| 2.66 (1.03, 4.3) * | 4.03 (2.77, 5.28) * | AM |  |  |  |  |  |  |
| 1.71 (-1.23, 4.67) | 3.08 (0.36, 5.8) * | -0.95 (-3.47, 1.58) | AT |  |  |  |  |  |
| -2.38 (-4, -0.74) * | -1.01 (-2.16, 0.13) | -5.04 (-5.58, -4.49) * | -4.09 (-6.56, -1.64) * | Control |  |  |  |  |
| -1.58 (-3.42, 0.28) * | -0.21 (-1.67, 1.24) | -4.24 (-5.28, -3.19) * | -3.29 (-5.91, -0.67) * | 0.8 (-0.09, 1.7) | EA |  |  |  |
| -2.6 (-4.43, -0.74) * | -1.23 (-2.67, 0.22) | -5.26 (-6.29, -4.22) * | -4.31 (-6.93, -1.7) * | -0.22 (-1.09, 0.66) | -1.02 (-1.88, -0.16) * | EA_CM |  |  |
| -2.35 (-4.63, -0.07) * | -0.99 (-2.96, 0.99) | -5.01 (-6.72, -3.31) * | -4.06 (-7.01, -1.13) * | 0.03 (-1.58, 1.64) | -0.77 (-2.63, 1.06) | 0.24 (-1.59, 2.08) | MB |  |
| -0.44 (-3.23, 2.38) | 0.93 (-1.62, 3.48) | -3.09 (-5.44, -0.77) * | -2.14 (-5.5, 1.18) | 1.94 (-0.32, 4.21) | 1.14 (-1.3, 3.57) | 2.16 (-0.27, 4.59) | 1.92 (-0.87, 4.68) | WA |

* Means P<0.05

**Table S3** FSH League table

| MD 95%CI | | | | | | | | | | | |
| --- | --- | --- | --- | --- | --- | --- | --- | --- | --- | --- | --- |
| AA |  |  |  |  |  |  |  |  |  |  |  |
| 0.61 (-0.15, 1.37) | AM |  |  |  |  |  |  |  |  |  |  |
| 6.14 (-12.25, 24.51) | 5.53 (-12.85, 23.93) | AM_CM |  |  |  |  |  |  |  |  |  |
| 0.91 (-0.18, 2) | 0.3 (-0.82, 1.41) | -5.24 (-23.62, 13.17) | AT |  |  |  |  |  |  |  |  |
| 6.27 (-1.04, 13.63) | 5.66 (-1.65, 13.02) | 0.12 (-19.58, 19.95) | 5.37 (-1.99, 12.77) | CA |  |  |  |  |  |  |  |
| -3.73 (-4.24, -3.23)* | -4.34 (-4.9, -3.79) * | -9.88 (-28.25, 8.52) | -4.64 (-5.61, -3.68) * | -10 (-17.34, -2.71) * | Control |  |  |  |  |  |  |
| -1.06 (-2.73, 0.6) | -1.67 (-3.35, 0.01) | -7.2 (-25.64, 11.25) | -1.97 (-3.82, -0.1) | -7.33 (-14.49, -0.23) | 2.67 (1.09, 4.26) * | EA |  |  |  |  |  |
| -3.7 (-15.92, 8.59) | -4.31 (-16.53, 7.98) | -9.82 (-31.94, 12.25) | -4.61 (-16.84, 7.71) | -9.98 (-24, 4.15) | 0.03 (-12.17, 12.34) | -2.64 (-14.74, 9.55) | HA |  |  |  |  |
| -3.77 (-5.24, -2.28) * | -4.37 (-5.85, -2.9) * | -9.9 (-28.33, 8.53) | -4.67 (-6.37, -2.98) * | -10.03 (-17.52, -2.61) * | -0.03 (-1.42, 1.36) | -2.7 (-4.81, -0.59) * | -0.07 (-12.43, 12.23) | MB |  |  |  |
| 4.22 (1.7, 6.72) * | 3.62 (1.08, 6.13) * | -1.92 (-20.5, 16.62) | 3.31 (0.65, 5.95) * | -2.05 (-9.81, 5.63) | -7.96 (5.49, 10.41) * | 5.28 (2.36, 8.2) * | 7.91 (-4.63, 20.35) | 7.98 (5.16, 10.8) * | PN |  |  |
| -8.16 (-12.82, -3.51) * | -8.77 (-13.42, -4.11) * | -14.29 (-33.33, 4.65) | -9.06 (-13.81, -4.32) | -14.43 (-23.12, -5.79) | -4.43 (-9.05, 0.2) | -7.1 (-12, -2.21) | -4.46 (-17.62, 8.65) | -4.39 (-9.22, 0.45) | -12.39 (-17.61, -7.12) * | SA |  |
| 0.8 (-0.9, 2.49) | 0.2 (-1.51, 1.9) | -5.34 (-23.78, 13.09) | -0.1 (-1.97, 1.77) | -5.46 (-12.98, 2) | 4.54 (2.92, 6.14) * | 1.87 (-0.4, 4.12) | 4.5 (-7.9, 16.84) | 4.57 (2.44, 6.71) * | -3.42 (-6.35, -0.49) | 8.97 (4.06, 13.87) * | WA |

* Means P<0.05

**Table S4** LH League table

| MD 95%CI | | | | | | | | | | | |
| --- | --- | --- | --- | --- | --- | --- | --- | --- | --- | --- | --- |
| AA |  |  |  |  |  |  |  |  |  |  |  |
| 1.68 (-1.54, 4.89) | AA_CM |  |  |  |  |  |  |  |  |  |  |
| -0.98 (-1.83, -0.13)* | -2.66 (-5.89, 0.57) | AM |  |  |  |  |  |  |  |  |  |
| 3.36 (-4.57, 11.34) | 1.68 (-6.8, 10.22) | 4.35 (-3.57, 12.32) | AM_CM |  |  |  |  |  |  |  |  |
| 0.19 (-0.92, 1.3) | -1.49 (-4.78, 1.8) | 1.17 (0.19, 2.15) * | -3.17 (-11.16, 4.75) | AT |  |  |  |  |  |  |  |
| 7.5 (4.82, 10.16) * | 5.82 (1.74, 9.91) * | 8.48 (5.86, 11.1) * | 4.14 (-4.2, 12.43) | 7.31 (4.62, 9.99) * | CA |  |  |  |  |  |  |
| -4.09 (-4.86, -3.32) * | -5.77 (-8.96, -2.57) * | -3.11 (-3.68, -2.53) * | -7.45 (-15.41, 0.44) | -4.28 (-5.09, -3.47) * | -11.58 (-14.14, -9.02) * | Control |  |  |  |  |  |
| -1.99 (-3.3, -0.7) * | -3.67 (-7.03, -0.31) * | -1.01 (-2.21, 0.17) | -5.36 (-13.37, 2.62) | -2.19 (-3.5, -0.86) * | -9.49 (-11.82, -7.15) * | 2.09 (1.05, 3.14) * | EA |  |  |  |  |
| 2.2 (-5.21, 9.58) | 0.52 (-7.5, 8.52) | 3.19 (-4.2, 10.52) | -1.17 (-11.92, 9.59) | 2.02 (-5.4, 9.39) | -5.29 (-12.95, 2.31) | 6.3 (-1.08, 13.62) | 4.2 (-3.11, 11.44) | HA |  |  |  |
| -0.92 (-2.68, 0.86) | -2.6 (-6.16, 0.96) | 0.06 (-1.55, 1.69) | -4.29 (-12.37, 3.78) | -1.11 (-2.92, 0.72) | -8.41 (-11.46, -5.37) * | 3.17 (1.54, 4.8) * | 1.08 (-0.86, 3.01) | -3.12 (-10.59, 4.43) | MB |  |  |
| -0.88 (-2.15, 0.38) | -2.56 (-5.91, 0.79) | 0.1 (-1.05, 1.25) | -4.25 (-12.26, 3.7) | -1.07 (-1.75, -0.39) * | -8.38 (-11.13, -5.62) * | 3.21 (2.2, 4.21) * | 1.11 (-0.34, 2.56) | -3.09 (-10.5, 4.34) | 0.04 (-1.88, 1.95) | SA |  |
| -0.24 (-1.48, 1) | -1.92 (-5.25, 1.42) | 0.74 (-0.39, 1.87) | -3.6 (-11.6, 4.35) | -0.43 (-1.7, 0.83) | -7.74 (-10.48, -5) * | 3.85 (2.88, 4.81) * | 1.75 (0.33, 3.18) * | -2.45 (-9.84, 4.97) | 0.68 (-1.21, 2.57) | 0.64 (-0.75, 2.03) | WA |

* Means P<0.05

**Table S5** E2 League table

| MD 95%CI | | | | | | | | | | | |
| --- | --- | --- | --- | --- | --- | --- | --- | --- | --- | --- | --- |
| AA |  |  |  |  |  |  |  |  |  |  |  |
| 0.36 (-0.95, 1.67) | AM |  |  |  |  |  |  |  |  |  |  |
| -9.32 (-21.56, 2.96) | -9.68 (-21.84, 2.58) | AM_CM |  |  |  |  |  |  |  |  |  |
| -2.66 (-4.12, -1.2)* | -3.02 (-3.99, -2.05) * | 6.67 (-5.6, 18.84) | AT |  |  |  |  |  |  |  |  |
| -4.17 (-13.35, 5.04) | -4.53 (-13.63, 4.63) | 5.18 (-10.06, 20.35) | -1.5 (-10.63, 7.67) | CA |  |  |  |  |  |  |  |
| 5.15 (3.94, 6.36) * | 4.8 (4.28, 5.31) * | 14.49 (2.24, 26.64) * | 7.81 (7, 8.63) * | 9.32 (0.17, 18.41) * | Control |  |  |  |  |  |  |
| 6.45 (4.03, 8.89) * | 6.09 (3.93, 8.27) * | 15.79 (3.36, 28.14) * | 9.11 (6.85, 11.37) * | 10.62 (1.75, 19.47) * | 1.3 (-0.81, 3.4) | EA |  |  |  |  |  |
| 34.03 (-5.72, 73.77) | 33.68 (-6.11, 73.41) | 43.32 (1.94, 85.03) | 36.71 (-3.1, 76.43) | 38.24 (-2.5, 78.8) | 28.9 (-10.87, 68.61) | 27.61 (-12.08, 67.26) | HA |  |  |  |  |
| -1.26 (-4.1, 1.55) | -1.62 (-4.23, 0.98) | 8.06 (-4.43, 20.49) | 1.4 (-1.3, 4.08) | 2.9 (-6.58, 12.37) | -6.42 (-8.99, -3.86) * | -7.71 (-11.04, -4.4) * | -35.32 (-75.11, 4.5) | MB |  |  |  |
| -21.76 (-32.22, -11.37) * | -22.11 (-32.51, -11.79) * | -12.41 (-28.41, 3.6) * | -19.09 (-29.5, -8.73) * | -17.6 (-31.38, -3.76) * | -26.9 (-37.29, -16.58) * | -28.21 (-38.82, -17.7) * | -55.73 (-96.92, -14.75) * | -20.5 (-31.2, -9.84) * | PN |  |  |
| -7.82 (-11.69, -3.92) * | -8.18 (-11.9, -4.44) * | 1.51 (-11.23, 14.26) | -5.16 (-8.89, -1.4) * | -3.65 (-13.48, 6.18) | -12.97 (-16.66, -9.26) * | -14.27 (-18.5, -10) * | -41.83 (-81.71, -1.88) * | -6.55 (-11.06, -2.04) * | 13.94 (2.94, 24.95) * | SA |  |
| 2.11 (0.42, 3.8) * | 1.76 (0.47, 3.04) * | 11.45 (-0.85, 23.64) | 4.78 (3.33, 6.21) * | 6.27 (-2.92, 15.45) | -3.04 (-4.22, -1.86) * | -4.34 (-6.75, -1.92) * | -31.96 (-71.69, 7.89) | 3.38 (0.56, 6.2) * | 23.86 (13.46, 34.34) * | 9.93 (6.04, 13.8) * | WA |

* Means P<0.05

**Table S6** Evaluation of the Certainty of evidence of Kupperman Using CINeMA Framework, mixed evidence.

| Comparison | Within-study bias | Reporting bias | Indirectness | Imprecision | Heterogeneity | Incoherence | Confidence rating | Reason(s) for downgrading |
| --- | --- | --- | --- | --- | --- | --- | --- | --- |
| AA:AM | Some concerns | Undetected | No concerns | Some concerns | Some concerns | No concerns | Low | ["Within-study bias","Heterogeneity"] |
| AA:Control | Some concerns | Undetected | No concerns | Major concerns | No concerns | No concerns | Low | ["Within-study bias","Imprecision"] |
| AM:Control | Some concerns | Undetected | No concerns | Some concerns | Some concerns | No concerns | Low | ["Within-study bias","Heterogeneity"] |
| AT:Control | Some concerns | Undetected | No concerns | Major concerns | No concerns | Major concerns | Very low | ["Within-study bias","Incoherence"] |
| Control:EA | Some concerns | Undetected | No concerns | Major concerns | No concerns | Major concerns | Very low | ["Within-study bias","Incoherence"] |
| Control:MB | Some concerns | Undetected | No concerns | Major concerns | No concerns | Major concerns | Very low | ["Within-study bias","Incoherence"] |
| Control:WA | Some concerns | Undetected | No concerns | Major concerns | No concerns | Major concerns | Very low | ["Within-study bias","Incoherence"] |
| AA:AT | Some concerns | Undetected | No concerns | Major concerns | No concerns | Major concerns | Very low | ["Within-study bias","Incoherence"] |
| AA:EA | Some concerns | Undetected | No concerns | Major concerns | No concerns | Major concerns | Very low | ["Within-study bias","Incoherence"] |
| AA:MB | Some concerns | Undetected | No concerns | Major concerns | No concerns | Major concerns | Very low | ["Within-study bias","Incoherence"] |
| AA:WA | Some concerns | Undetected | No concerns | Major concerns | No concerns | Major concerns | Very low | ["Within-study bias","Incoherence"] |
| AM:AT | Some concerns | Undetected | No concerns | Major concerns | No concerns | Major concerns | Very low | ["Within-study bias","Incoherence"] |
| AM:EA | Some concerns | Undetected | No concerns | Major concerns | No concerns | Major concerns | Very low | ["Within-study bias","Incoherence"] |
| AM:MB | Some concerns | Undetected | No concerns | Major concerns | No concerns | Major concerns | Very low | ["Within-study bias","Incoherence"] |
| AM:WA | Some concerns | Undetected | No concerns | Major concerns | No concerns | Major concerns | Very low | ["Within-study bias","Incoherence"] |
| AT:EA | Some concerns | Undetected | No concerns | Major concerns | No concerns | Major concerns | Very low | ["Within-study bias","Incoherence"] |
| AT:MB | Some concerns | Undetected | No concerns | Major concerns | No concerns | Major concerns | Very low | ["Within-study bias","Incoherence"] |
| AT:WA | Some concerns | Undetected | No concerns | Major concerns | No concerns | Major concerns | Very low | ["Within-study bias","Incoherence"] |
| EA:MB | Some concerns | Undetected | No concerns | Major concerns | No concerns | Major concerns | Very low | ["Within-study bias","Incoherence"] |
| EA:WA | Some concerns | Undetected | No concerns | Major concerns | No concerns | Major concerns | Very low | ["Within-study bias","Incoherence"] |
| MB:WA | Some concerns | Undetected | No concerns | Major concerns | No concerns | Major concerns | Very low | ["Within-study bias","Incoherence"] |

**Table S7** Evaluation of the Certainty of evidence of FSH Using CINeMA Framework, mixed evidence.

| Comparison | Number of studies | Within-study bias | Reporting bias | Indirectness | Imprecision | Heterogeneity | Incoherence | Confidence rating | Reason(s) for downgrading |
| --- | --- | --- | --- | --- | --- | --- | --- | --- | --- |
| AA:Control | 4 | Some concerns | Undetected | No concerns | No concerns | Some concerns | Major concerns | Very low | ["Within-study bias","Incoherence"] |
| AM:Control | 7 | Some concerns | Undetected | No concerns | No concerns | No concerns | Major concerns | low | ["Within-study bias","Incoherence"] |
| AM:UM | 1 | Some concerns | Undetected | No concerns | Some concerns | No concerns | Major concerns | Very low | ["Within-study bias","Incoherence"] |
| AMandCM:Control | 1 | Some concerns | Undetected | No concerns | Major concerns | No concerns | Major concerns | Very low | ["Within-study bias","Incoherence"] |
| AT:Control | 4 | Some concerns | Undetected | No concerns | No concerns | Some concerns | Major concerns | Very low | ["Within-study bias","Incoherence"] |
| CA:EA | 1 | Some concerns | Undetected | No concerns | Some concerns | No concerns | Major concerns | Very low | ["Within-study bias","Incoherence"] |
| Control:EA | 4 | Some concerns | Undetected | No concerns | No concerns | Major concerns | Major concerns | Very low | ["Within-study bias","Incoherence"] |
| Control:MB | 1 | Some concerns | Undetected | No concerns | Some concerns | Some concerns | Major concerns | Very low | ["Within-study bias","Incoherence"] |
| Control:PN | 1 | Some concerns | Undetected | No concerns | Some concerns | No concerns | Major concerns | Very low | ["Within-study bias","Incoherence"] |
| Control:SA | 1 | Some concerns | Undetected | No concerns | Some concerns | Some concerns | Major concerns | Very low | ["Within-study bias","Incoherence"] |
| Control:WA | 4 | Some concerns | Undetected | No concerns | No concerns | Some concerns | Major concerns | Very low | ["Within-study bias","Incoherence"] |
| EA:HA | 2 | Some concerns | Undetected | No concerns | Major concerns | No concerns | Major concerns | Very low | ["Within-study bias","Incoherence"] |
| EA:MC | 1 | Some concerns | Undetected | No concerns | Major concerns | No concerns | Major concerns | Very low | ["Within-study bias","Incoherence"] |
| RA:SA | 1 | No concerns | Undetected | No concerns | Some concerns | Some concerns | Major concerns | low | ["Incoherence"] |
| AA:AM | 0 | Some concerns | Undetected | No concerns | No concerns | No concerns | Major concerns | low | ["Within-study bias","Incoherence"] |
| AA:AMandCM | 0 | Some concerns | Undetected | No concerns | Some concerns | Some concerns | Major concerns | Very low | ["Within-study bias","Incoherence"] |
| AA:AT | 0 | Some concerns | Undetected | No concerns | No concerns | Some concerns | Major concerns | Very low | ["Within-study bias","Incoherence"] |
| AA:CA | 0 | Some concerns | Undetected | No concerns | Some concerns | No concerns | Major concerns | Very low | ["Within-study bias","Incoherence"] |
| AA:EA | 0 | Some concerns | Undetected | No concerns | Some concerns | No concerns | Major concerns | Very low | ["Within-study bias","Incoherence"] |
| AA:HA | 0 | Some concerns | Undetected | No concerns | Major concerns | No concerns | Major concerns | Very low | ["Within-study bias","Incoherence"] |
| AA:MB | 0 | Some concerns | Undetected | No concerns | Some concerns | Some concerns | Major concerns | Very low | ["Within-study bias","Incoherence"] |
| AA:MC | 0 | Some concerns | Undetected | No concerns | Major concerns | No concerns | Major concerns | Very low | ["Within-study bias","Incoherence"] |
| AA:PN | 0 | Some concerns | Undetected | No concerns | Some concerns | No concerns | Major concerns | Very low | ["Within-study bias","Incoherence"] |
| AA:RA | 0 | Some concerns | Undetected | No concerns | Major concerns | No concerns | Major concerns | Very low | ["Within-study bias","Incoherence"] |
| AA:SA | 0 | Some concerns | Undetected | No concerns | Major concerns | No concerns | Major concerns | Very low | ["Within-study bias","Incoherence"] |
| AA:UM | 0 | Some concerns | Undetected | No concerns | Some concerns | No concerns | Major concerns | Very low | ["Within-study bias","Incoherence"] |
| AA:WA | 0 | Some concerns | Undetected | No concerns | Some concerns | No concerns | Major concerns | Very low | ["Within-study bias","Incoherence"] |
| AM:AMandCM | 0 | Some concerns | Undetected | No concerns | Major concerns | No concerns | Major concerns | Very low | ["Within-study bias","Incoherence"] |
| AM:AT | 0 | Some concerns | Undetected | No concerns | No concerns | Some concerns | Major concerns | Very low | ["Within-study bias","Incoherence"] |
| AM:CA | 0 | Some concerns | Undetected | No concerns | Major concerns | No concerns | Major concerns | Very low | ["Within-study bias","Incoherence"] |
| AM:EA | 0 | Some concerns | Undetected | No concerns | No concerns | Some concerns | Major concerns | Very low | ["Within-study bias","Incoherence"] |
| AM:HA | 0 | Some concerns | Undetected | No concerns | Some concerns | No concerns | Major concerns | Very low | ["Within-study bias","Incoherence"] |
| AM:MB | 0 | Some concerns | Undetected | No concerns | No concerns | Some concerns | Major concerns | Very low | ["Within-study bias","Incoherence"] |
| AM:MC | 0 | Some concerns | Undetected | No concerns | Some concerns | No concerns | Major concerns | Very low | ["Within-study bias","Incoherence"] |
| AM:PN | 0 | Some concerns | Undetected | No concerns | Some concerns | Some concerns | Major concerns | Very low | ["Within-study bias","Incoherence"] |
| AM:RA | 0 | Some concerns | Undetected | No concerns | No concerns | Some concerns | Major concerns | Very low | ["Within-study bias","Incoherence"] |
| AM:SA | 0 | Some concerns | Undetected | No concerns | No concerns | No concerns | Major concerns | low | ["Within-study bias","Incoherence"] |
| AM:WA | 0 | Some concerns | Undetected | No concerns | No concerns | Some concerns | Major concerns | Very low | ["Within-study bias","Incoherence"] |
| AMandCM:AT | 0 | Some concerns | Undetected | No concerns | Major concerns | No concerns | Major concerns | Very low | ["Within-study bias","Incoherence"] |
| AMandCM:CA | 0 | Some concerns | Undetected | No concerns | Major concerns | No concerns | Major concerns | Very low | ["Within-study bias","Incoherence"] |
| AMandCM:EA | 0 | Some concerns | Undetected | No concerns | Major concerns | No concerns | Major concerns | Very low | ["Within-study bias","Incoherence"] |
| AMandCM:HA | 0 | Some concerns | Undetected | No concerns | Major concerns | No concerns | Major concerns | Very low | ["Within-study bias","Incoherence"] |
| AMandCM:MB | 0 | Some concerns | Undetected | No concerns | Major concerns | No concerns | Major concerns | Very low | ["Within-study bias","Incoherence"] |
| AMandCM:MC | 0 | Some concerns | Undetected | No concerns | Major concerns | No concerns | Major concerns | Very low | ["Within-study bias","Incoherence"] |
| AMandCM:PN | 0 | Some concerns | Undetected | No concerns | Major concerns | No concerns | Major concerns | Very low | ["Within-study bias","Incoherence"] |
| AMandCM:RA | 0 | Some concerns | Undetected | No concerns | Major concerns | No concerns | Major concerns | Very low | ["Within-study bias","Incoherence"] |
| AMandCM:SA | 0 | Some concerns | Undetected | No concerns | Some concerns | Some concerns | Major concerns | Very low | ["Within-study bias","Incoherence"] |
| AMandCM:UM | 0 | Some concerns | Undetected | No concerns | Major concerns | No concerns | Major concerns | Very low | ["Within-study bias","Incoherence"] |
| AMandCM:WA | 0 | Some concerns | Undetected | No concerns | Major concerns | No concerns | Major concerns | Very low | ["Within-study bias","Incoherence"] |
| AT:CA | 0 | Some concerns | Undetected | No concerns | Major concerns | No concerns | Major concerns | Very low | ["Within-study bias","Incoherence"] |
| AT:EA | 0 | Some concerns | Undetected | No concerns | Some concerns | No concerns | Major concerns | Very low | ["Within-study bias","Incoherence"] |
| AT:HA | 0 | Some concerns | Undetected | No concerns | Some concerns | Some concerns | Major concerns | Very low | ["Within-study bias","Incoherence"] |
| AT:MB | 0 | Some concerns | Undetected | No concerns | Some concerns | Some concerns | Major concerns | Very low | ["Within-study bias","Incoherence"] |
| AT:MC | 0 | Some concerns | Undetected | No concerns | Major concerns | No concerns | Major concerns | Very low | ["Within-study bias","Incoherence"] |
| AT:PN | 0 | Some concerns | Undetected | No concerns | Some concerns | Some concerns | Major concerns | Very low | ["Within-study bias","Incoherence"] |
| AT:RA | 0 | Some concerns | Undetected | No concerns | Some concerns | No concerns | Major concerns | Very low | ["Within-study bias","Incoherence"] |
| AT:SA | 0 | Some concerns | Undetected | No concerns | Some concerns | No concerns | Major concerns | Very low | ["Within-study bias","Incoherence"] |
| AT:UM | 0 | Some concerns | Undetected | No concerns | Major concerns | No concerns | Major concerns | Very low | ["Within-study bias","Incoherence"] |
| AT:WA | 0 | Some concerns | Undetected | No concerns | No concerns | Major concerns | Major concerns | Very low | ["Within-study bias","Incoherence"] |
| CA:Control | 0 | Some concerns | Undetected | No concerns | Some concerns | No concerns | Major concerns | Very low | ["Within-study bias","Incoherence"] |
| CA:HA | 0 | Some concerns | Undetected | No concerns | Some concerns | Some concerns | Major concerns | Very low | ["Within-study bias","Incoherence"] |
| CA:MB | 0 | Some concerns | Undetected | No concerns | Some concerns | Some concerns | Major concerns | Very low | ["Within-study bias","Incoherence"] |
| CA:MC | 0 | Some concerns | Undetected | No concerns | Major concerns | No concerns | Major concerns | Very low | ["Within-study bias","Incoherence"] |
| CA:PN | 0 | Some concerns | Undetected | No concerns | Major concerns | No concerns | Major concerns | Very low | ["Within-study bias","Incoherence"] |
| CA:RA | 0 | Some concerns | Undetected | No concerns | Some concerns | Some concerns | Major concerns | Very low | ["Within-study bias","Incoherence"] |
| CA:SA | 0 | Some concerns | Undetected | No concerns | Some concerns | No concerns | Major concerns | Very low | ["Within-study bias","Incoherence"] |
| CA:UM | 0 | Some concerns | Undetected | No concerns | Major concerns | No concerns | Major concerns | Very low | ["Within-study bias","Incoherence"] |
| CA:WA | 0 | Some concerns | Undetected | No concerns | Some concerns | Some concerns | Major concerns | Very low | ["Within-study bias","Incoherence"] |
| Control:HA | 0 | Some concerns | Undetected | No concerns | Major concerns | No concerns | Major concerns | Very low | ["Within-study bias","Incoherence"] |
| Control:MC | 0 | Some concerns | Undetected | No concerns | Major concerns | No concerns | Major concerns | Very low | ["Within-study bias","Incoherence"] |
| Control:RA | 0 | Some concerns | Undetected | No concerns | Major concerns | No concerns | Major concerns | Very low | ["Within-study bias","Incoherence"] |
| Control:UM | 0 | Some concerns | Undetected | No concerns | Some concerns | Some concerns | Major concerns | Very low | ["Within-study bias","Incoherence"] |
| EA:MB | 0 | Some concerns | Undetected | No concerns | Major concerns | No concerns | Major concerns | Very low | ["Within-study bias","Incoherence"] |
| EA:PN | 0 | Some concerns | Undetected | No concerns | Some concerns | Some concerns | Major concerns | Very low | ["Within-study bias","Incoherence"] |
| EA:RA | 0 | Some concerns | Undetected | No concerns | Some concerns | Some concerns | Major concerns | Very low | ["Within-study bias","Incoherence"] |
| EA:SA | 0 | Some concerns | Undetected | No concerns | Some concerns | Some concerns | Major concerns | Very low | ["Within-study bias","Incoherence"] |
| EA:UM | 0 | Some concerns | Undetected | No concerns | Some concerns | Some concerns | Major concerns | Very low | ["Within-study bias","Incoherence"] |
| EA:WA | 0 | Some concerns | Undetected | No concerns | Some concerns | Some concerns | Major concerns | Very low | ["Within-study bias","Incoherence"] |
| HA:MB | 0 | Some concerns | Undetected | No concerns | Major concerns | No concerns | Major concerns | Very low | ["Within-study bias","Incoherence"] |
| HA:MC | 0 | Some concerns | Undetected | No concerns | Major concerns | No concerns | Major concerns | Very low | ["Within-study bias","Incoherence"] |
| HA:PN | 0 | Some concerns | Undetected | No concerns | Some concerns | Some concerns | Major concerns | Very low | ["Within-study bias","Incoherence"] |
| HA:RA | 0 | Some concerns | Undetected | No concerns | Major concerns | No concerns | Major concerns | Very low | ["Within-study bias","Incoherence"] |
| HA:SA | 0 | Some concerns | Undetected | No concerns | Major concerns | No concerns | Major concerns | Very low | ["Within-study bias","Incoherence"] |
| HA:UM | 0 | Some concerns | Undetected | No concerns | Major concerns | No concerns | Major concerns | Very low | ["Within-study bias","Incoherence"] |
| HA:WA | 0 | Some concerns | Undetected | No concerns | Major concerns | No concerns | Major concerns | Very low | ["Within-study bias","Incoherence"] |
| MB:MC | 0 | Some concerns | Undetected | No concerns | Major concerns | No concerns | Major concerns | Very low | ["Within-study bias","Incoherence"] |
| MB:PN | 0 | Some concerns | Undetected | No concerns | Some concerns | Some concerns | Major concerns | Very low | ["Within-study bias","Incoherence"] |
| MB:RA | 0 | Some concerns | Undetected | No concerns | Major concerns | No concerns | Major concerns | Very low | ["Within-study bias","Incoherence"] |
| MB:SA | 0 | Some concerns | Undetected | No concerns | Major concerns | No concerns | Major concerns | Very low | ["Within-study bias","Incoherence"] |
| MB:UM | 0 | Some concerns | Undetected | No concerns | Major concerns | No concerns | Major concerns | Very low | ["Within-study bias","Incoherence"] |
| MB:WA | 0 | Some concerns | Undetected | No concerns | Some concerns | Some concerns | Major concerns | Very low | ["Within-study bias","Incoherence"] |
| MC:PN | 0 | Some concerns | Undetected | No concerns | Major concerns | No concerns | Major concerns | Very low | ["Within-study bias","Incoherence"] |
| MC:RA | 0 | Some concerns | Undetected | No concerns | Major concerns | No concerns | Major concerns | Very low | ["Within-study bias","Incoherence"] |
| MC:SA | 0 | Some concerns | Undetected | No concerns | Major concerns | No concerns | Major concerns | Very low | ["Within-study bias","Incoherence"] |
| MC:UM | 0 | Some concerns | Undetected | No concerns | Major concerns | No concerns | Major concerns | Very low | ["Within-study bias","Incoherence"] |
| MC:WA | 0 | Some concerns | Undetected | No concerns | Major concerns | No concerns | Major concerns | Very low | ["Within-study bias","Incoherence"] |
| PN:RA | 0 | Some concerns | Undetected | No concerns | Some concerns | No concerns | Major concerns | Very low | ["Within-study bias","Incoherence"] |
| PN:SA | 0 | Some concerns | Undetected | No concerns | Some concerns | No concerns | Major concerns | Very low | ["Within-study bias","Incoherence"] |
| PN:UM | 0 | Some concerns | Undetected | No concerns | Major concerns | No concerns | Major concerns | Very low | ["Within-study bias","Incoherence"] |
| PN:WA | 0 | Some concerns | Undetected | No concerns | Some concerns | Some concerns | Major concerns | Very low | ["Within-study bias","Incoherence"] |
| RA:UM | 0 | Some concerns | Undetected | No concerns | Some concerns | Some concerns | Major concerns | Very low | ["Within-study bias","Incoherence"] |
| RA:WA | 0 | Some concerns | Undetected | No concerns | Some concerns | Some concerns | Major concerns | Very low | ["Within-study bias","Incoherence"] |
| SA:UM | 0 | Some concerns | Undetected | No concerns | Some concerns | Some concerns | Major concerns | Very low | ["Within-study bias","Incoherence"] |
| SA:WA | 0 | Some concerns | Undetected | No concerns | Some concerns | No concerns | Major concerns | Very low | ["Within-study bias","Incoherence"] |
| UM:WA | 0 | Some concerns | Undetected | No concerns | Major concerns | No concerns | Major concerns | Very low | ["Within-study bias","Incoherence"] |

**Table S8** Evaluation of the Certainty of evidence of E2 Using CINeMA Framework, mixed evidence.

| Comparison | Number of studies | Within-study bias | Reporting bias | Indirectness | Imprecision | Heterogeneity | Incoherence | Confidence rating | Reason(s) for downgrading |
| --- | --- | --- | --- | --- | --- | --- | --- | --- | --- |
| AA:Control | 4 | Some concerns | Undetected | No concerns | Some concerns | Some concerns | Major concerns | Very low | ["Within-study bias", "Incoherence"] |
| AM:Control | 7 | Some concerns | Undetected | No concerns | No concerns | Some concerns | Major concerns | Very low | ["Within-study bias", "Incoherence"] |
| AM:UM | 1 | Some concerns | Undetected | No concerns | Some concerns | Some concerns | Major concerns | Very low | ["Within-study bias", "Incoherence"] |
| AMandCM:Control | 1 | Some concerns | Undetected | No concerns | Some concerns | Some concerns | Major concerns | Very low | ["Within-study bias", "Incoherence"] |
| AT:Control | 4 | Some concerns | Undetected | No concerns | No concerns | Major concerns | Major concerns | Very low | ["Within-study bias", "Incoherence"] |
| CA:EA | 1 | Some concerns | Undetected | No concerns | Major concerns | No concerns | Major concerns | Very low | ["Within-study bias", "Incoherence"] |
| Control:EA | 5 | Some concerns | Undetected | No concerns | Some concerns | Some concerns | Major concerns | Very low | ["Within-study bias", "Incoherence"] |
| Control:MB | 1 | Some concerns | Undetected | No concerns | Major concerns | No concerns | Major concerns | Very low | ["Within-study bias", "Incoherence"] |
| Control:PN | 1 | Some concerns | Undetected | No concerns | No concerns | No concerns | Major concerns | low | ["Within-study bias", "Incoherence"] |
| Control:SA | 1 | Some concerns | Undetected | No concerns | Major concerns | No concerns | Major concerns | Very low | ["Within-study bias", "Incoherence"] |
| Control:WA | 4 | Some concerns | Undetected | No concerns | Some concerns | Some concerns | Major concerns | Very low | ["Within-study bias", "Incoherence"] |
| EA:HA | 2 | Some concerns | Undetected | No concerns | Major concerns | No concerns | Major concerns | Very low | ["Within-study bias", "Incoherence"] |
| EA:MC | 1 | Some concerns | Undetected | No concerns | Major concerns | No concerns | Major concerns | Very low | ["Within-study bias", "Incoherence"] |
| RA:SA | 1 | No concerns | Undetected | No concerns | Some concerns | Some concerns | Major concerns | low | [ "Incoherence"] |
| AA:AM | 0 | Some concerns | Undetected | No concerns | Some concerns | Some concerns | Major concerns | Very low | ["Within-study bias", "Incoherence"] |
| AA:AMandCM | 0 | Some concerns | Undetected | No concerns | Major concerns | No concerns | Major concerns | Very low | ["Within-study bias", "Incoherence"] |
| AA:AT | 0 | Some concerns | Undetected | No concerns | Major concerns | No concerns | Major concerns | Very low | ["Within-study bias", "Incoherence"] |
| AA:CA | 0 | Some concerns | Undetected | No concerns | Major concerns | No concerns | Major concerns | Very low | ["Within-study bias", "Incoherence"] |
| AA:EA | 0 | Some concerns | Undetected | No concerns | Major concerns | No concerns | Major concerns | Very low | ["Within-study bias", "Incoherence"] |
| AA:HA | 0 | Some concerns | Undetected | No concerns | Major concerns | No concerns | Major concerns | Very low | ["Within-study bias", "Incoherence"] |
| AA:MB | 0 | Some concerns | Undetected | No concerns | Major concerns | No concerns | Major concerns | Very low | ["Within-study bias", "Incoherence"] |
| AA:MC | 0 | Some concerns | Undetected | No concerns | Major concerns | No concerns | Major concerns | Very low | ["Within-study bias", "Incoherence"] |
| AA:PN | 0 | Some concerns | Undetected | No concerns | No concerns | Some concerns | Major concerns | Very low | ["Within-study bias", "Incoherence"] |
| AA:RA | 0 | Some concerns | Undetected | No concerns | Major concerns | No concerns | Major concerns | Very low | ["Within-study bias", "Incoherence"] |
| AA:SA | 0 | Some concerns | Undetected | No concerns | Major concerns | No concerns | Major concerns | Very low | ["Within-study bias", "Incoherence"] |
| AA:UM | 0 | Some concerns | Undetected | No concerns | Major concerns | No concerns | Major concerns | Very low | ["Within-study bias", "Incoherence"] |
| AA:WA | 0 | Some concerns | Undetected | No concerns | Major concerns | No concerns | Major concerns | Very low | ["Within-study bias", "Incoherence"] |
| AM:AMandCM | 0 | Some concerns | Undetected | No concerns | Major concerns | No concerns | Major concerns | Very low | ["Within-study bias", "Incoherence"] |
| AM:AT | 0 | Some concerns | Undetected | No concerns | Major concerns | No concerns | Major concerns | Very low | ["Within-study bias", "Incoherence"] |
| AM:CA | 0 | Some concerns | Undetected | No concerns | Major concerns | No concerns | Major concerns | Very low | ["Within-study bias", "Incoherence"] |
| AM:EA | 0 | Some concerns | Undetected | No concerns | No concerns | Major concerns | Major concerns | Very low | ["Within-study bias", "Incoherence"] |
| AM:HA | 0 | Some concerns | Undetected | No concerns | Major concerns | No concerns | Major concerns | Very low | ["Within-study bias", "Incoherence"] |
| AM:MB | 0 | Some concerns | Undetected | No concerns | Major concerns | No concerns | Major concerns | Very low | ["Within-study bias", "Incoherence"] |
| AM:MC | 0 | Some concerns | Undetected | No concerns | Major concerns | No concerns | Major concerns | Very low | ["Within-study bias", "Incoherence"] |
| AM:PN | 0 | Some concerns | Undetected | No concerns | Major concerns | No concerns | Major concerns | Very low | ["Within-study bias", "Incoherence"] |
| AM:RA | 0 | Some concerns | Undetected | No concerns | Some concerns | Some concerns | Major concerns | Very low | ["Within-study bias", "Incoherence"] |
| AM:SA | 0 | Some concerns | Undetected | No concerns | Major concerns | No concerns | Major concerns | Very low | ["Within-study bias", "Incoherence"] |
| AM:WA | 0 | Some concerns | Undetected | No concerns | Some concerns | Some concerns | Major concerns | Very low | ["Within-study bias", "Incoherence"] |
| AMandCM:AT | 0 | Some concerns | Undetected | No concerns | Major concerns | No concerns | Major concerns | Very low | ["Within-study bias", "Incoherence"] |
| AMandCM:CA | 0 | Some concerns | Undetected | No concerns | Major concerns | No concerns | Major concerns | Very low | ["Within-study bias", "Incoherence"] |
| AMandCM:EA | 0 | Some concerns | Undetected | No concerns | Major concerns | No concerns | Major concerns | Very low | ["Within-study bias", "Incoherence"] |
| AMandCM:HA | 0 | Some concerns | Undetected | No concerns | Major concerns | No concerns | Major concerns | Very low | ["Within-study bias", "Incoherence"] |
| AMandCM:MB | 0 | Some concerns | Undetected | No concerns | Major concerns | No concerns | Major concerns | Very low | ["Within-study bias", "Incoherence"] |
| AMandCM:MC | 0 | Some concerns | Undetected | No concerns | Major concerns | No concerns | Major concerns | Very low | ["Within-study bias", "Incoherence"] |
| AMandCM:PN | 0 | Some concerns | Undetected | No concerns | Major concerns | No concerns | Major concerns | Very low | ["Within-study bias", "Incoherence"] |
| AMandCM:RA | 0 | Some concerns | Undetected | No concerns | Major concerns | No concerns | Major concerns | Very low | ["Within-study bias", "Incoherence"] |
| AMandCM:SA | 0 | Some concerns | Undetected | No concerns | Major concerns | No concerns | Major concerns | Very low | ["Within-study bias", "Incoherence"] |
| AMandCM:UM | 0 | Some concerns | Undetected | No concerns | Major concerns | No concerns | Major concerns | Very low | ["Within-study bias", "Incoherence"] |
| AMandCM:WA | 0 | Some concerns | Undetected | No concerns | Major concerns | No concerns | Major concerns | Very low | ["Within-study bias", "Incoherence"] |
| AT:CA | 0 | Some concerns | Undetected | No concerns | Major concerns | No concerns | Major concerns | Very low | ["Within-study bias", "Incoherence"] |
| AT:EA | 0 | Some concerns | Undetected | No concerns | Some concerns | Some concerns | Major concerns | Very low | ["Within-study bias", "Incoherence"] |
| AT:HA | 0 | Some concerns | Undetected | No concerns | Major concerns | No concerns | Major concerns | Very low | ["Within-study bias", "Incoherence"] |
| AT:MB | 0 | Some concerns | Undetected | No concerns | Major concerns | No concerns | Major concerns | Very low | ["Within-study bias", "Incoherence"] |
| AT:MC | 0 | Some concerns | Undetected | No concerns | Major concerns | No concerns | Major concerns | Very low | ["Within-study bias", "Incoherence"] |
| AT:PN | 0 | Some concerns | Undetected | No concerns | Some concerns | Some concerns | Major concerns | Very low | ["Within-study bias", "Incoherence"] |
| AT:RA | 0 | Some concerns | Undetected | No concerns | Major concerns | No concerns | Major concerns | Very low | ["Within-study bias", "Incoherence"] |
| AT:SA | 0 | Some concerns | Undetected | No concerns | Major concerns | No concerns | Major concerns | Very low | ["Within-study bias", "Incoherence"] |
| AT:UM | 0 | Some concerns | Undetected | No concerns | Major concerns | No concerns | Major concerns | Very low | ["Within-study bias", "Incoherence"] |
| AT:WA | 0 | Some concerns | Undetected | No concerns | Major concerns | No concerns | Major concerns | Very low | ["Within-study bias", "Incoherence"] |
| CA:Control | 0 | Some concerns | Undetected | No concerns | Some concerns | Some concerns | Major concerns | Very low | ["Within-study bias", "Incoherence"] |
| CA:HA | 0 | Some concerns | Undetected | No concerns | Major concerns | No concerns | Major concerns | Very low | ["Within-study bias", "Incoherence"] |
| CA:MB | 0 | Some concerns | Undetected | No concerns | Major concerns | No concerns | Major concerns | Very low | ["Within-study bias", "Incoherence"] |
| CA:MC | 0 | Some concerns | Undetected | No concerns | Major concerns | No concerns | Major concerns | Very low | ["Within-study bias", "Incoherence"] |
| CA:PN | 0 | Some concerns | Undetected | No concerns | Major concerns | No concerns | Major concerns | Very low | ["Within-study bias", "Incoherence"] |
| CA:RA | 0 | Some concerns | Undetected | No concerns | Major concerns | No concerns | Major concerns | Very low | ["Within-study bias", "Incoherence"] |
| CA:SA | 0 | Some concerns | Undetected | No concerns | Major concerns | No concerns | Major concerns | Very low | ["Within-study bias", "Incoherence"] |
| CA:UM | 0 | Some concerns | Undetected | No concerns | Major concerns | No concerns | Major concerns | Very low | ["Within-study bias", "Incoherence"] |
| CA:WA | 0 | Some concerns | Undetected | No concerns | Major concerns | No concerns | Major concerns | Very low | ["Within-study bias", "Incoherence"] |
| Control:HA | 0 | Some concerns | Undetected | No concerns | Major concerns | No concerns | Major concerns | Very low | ["Within-study bias", "Incoherence"] |
| Control:MC | 0 | Some concerns | Undetected | No concerns | Major concerns | No concerns | Major concerns | Very low | ["Within-study bias", "Incoherence"] |
| Control:RA | 0 | Some concerns | Undetected | No concerns | Major concerns | No concerns | Major concerns | Very low | ["Within-study bias", "Incoherence"] |
| Control:UM | 0 | Some concerns | Undetected | No concerns | Major concerns | No concerns | Major concerns | Very low | ["Within-study bias", "Incoherence"] |
| EA:MB | 0 | Some concerns | Undetected | No concerns | Major concerns | No concerns | Major concerns | Very low | ["Within-study bias", "Incoherence"] |
| EA:PN | 0 | Some concerns | Undetected | No concerns | No concerns | Some concerns | Major concerns | Very low | ["Within-study bias", "Incoherence"] |
| EA:RA | 0 | Some concerns | Undetected | No concerns | Major concerns | No concerns | Major concerns | Very low | ["Within-study bias", "Incoherence"] |
| EA:SA | 0 | Some concerns | Undetected | No concerns | Major concerns | No concerns | Major concerns | Very low | ["Within-study bias", "Incoherence"] |
| EA:UM | 0 | Some concerns | Undetected | No concerns | Major concerns | No concerns | Major concerns | Very low | ["Within-study bias", "Incoherence"] |
| EA:WA | 0 | Some concerns | Undetected | No concerns | Major concerns | No concerns | Major concerns | Very low | ["Within-study bias", "Incoherence"] |
| HA:MB | 0 | Some concerns | Undetected | No concerns | Major concerns | No concerns | Major concerns | Very low | ["Within-study bias", "Incoherence"] |
| HA:MC | 0 | Some concerns | Undetected | No concerns | Major concerns | No concerns | Major concerns | Very low | ["Within-study bias", "Incoherence"] |
| HA:PN | 0 | Some concerns | Undetected | No concerns | No concerns | Some concerns | Major concerns | Very low | ["Within-study bias", "Incoherence"] |
| HA:RA | 0 | Some concerns | Undetected | No concerns | Major concerns | No concerns | Major concerns | Very low | ["Within-study bias", "Incoherence"] |
| HA:SA | 0 | Some concerns | Undetected | No concerns | Major concerns | No concerns | Major concerns | Very low | ["Within-study bias", "Incoherence"] |
| HA:UM | 0 | Some concerns | Undetected | No concerns | Major concerns | No concerns | Major concerns | Very low | ["Within-study bias", "Incoherence"] |
| HA:WA | 0 | Some concerns | Undetected | No concerns | Major concerns | No concerns | Major concerns | Very low | ["Within-study bias", "Incoherence"] |
| MB:MC | 0 | Some concerns | Undetected | No concerns | Major concerns | No concerns | Major concerns | Very low | ["Within-study bias", "Incoherence"] |
| MB:PN | 0 | Some concerns | Undetected | No concerns | Major concerns | No concerns | Major concerns | Very low | ["Within-study bias", "Incoherence"] |
| MB:RA | 0 | Some concerns | Undetected | No concerns | Major concerns | No concerns | Major concerns | Very low | ["Within-study bias", "Incoherence"] |
| MB:SA | 0 | Some concerns | Undetected | No concerns | Major concerns | No concerns | Major concerns | Very low | ["Within-study bias", "Incoherence"] |
| MB:UM | 0 | Some concerns | Undetected | No concerns | Major concerns | No concerns | Major concerns | Very low | ["Within-study bias", "Incoherence"] |
| MB:WA | 0 | Some concerns | Undetected | No concerns | Major concerns | No concerns | Major concerns | Very low | ["Within-study bias", "Incoherence"] |
| MC:PN | 0 | Some concerns | Undetected | No concerns | Major concerns | No concerns | Major concerns | Very low | ["Within-study bias", "Incoherence"] |
| MC:RA | 0 | Some concerns | Undetected | No concerns | Major concerns | No concerns | Major concerns | Very low | ["Within-study bias", "Incoherence"] |
| MC:SA | 0 | Some concerns | Undetected | No concerns | Major concerns | No concerns | Major concerns | Very low | ["Within-study bias", "Incoherence"] |
| MC:UM | 0 | Some concerns | Undetected | No concerns | Major concerns | No concerns | Major concerns | Very low | ["Within-study bias", "Incoherence"] |
| MC:WA | 0 | Some concerns | Undetected | No concerns | Major concerns | No concerns | Major concerns | Very low | ["Within-study bias", "Incoherence"] |
| PN:RA | 0 | Some concerns | Undetected | No concerns | No concerns | Some concerns | Major concerns | Very low | ["Within-study bias", "Incoherence"] |
| PN:SA | 0 | Some concerns | Undetected | No concerns | Major concerns | No concerns | Major concerns | Very low | ["Within-study bias", "Incoherence"] |
| PN:UM | 0 | Some concerns | Undetected | No concerns | No concerns | Some concerns | Major concerns | Very low | ["Within-study bias", "Incoherence"] |
| PN:WA | 0 | Some concerns | Undetected | No concerns | No concerns | Some concerns | Major concerns | Very low | ["Within-study bias", "Incoherence"] |
| RA:UM | 0 | Some concerns | Undetected | No concerns | Major concerns | No concerns | Major concerns | Very low | ["Within-study bias", "Incoherence"] |
| RA:WA | 0 | Some concerns | Undetected | No concerns | Major concerns | No concerns | Major concerns | Very low | ["Within-study bias", "Incoherence"] |
| SA:UM | 0 | Some concerns | Undetected | No concerns | Major concerns | No concerns | Major concerns | Very low | ["Within-study bias", "Incoherence"] |
| SA:WA | 0 | Some concerns | Undetected | No concerns | Major concerns | No concerns | Major concerns | Very low | ["Within-study bias", "Incoherence"] |
| UM:WA | 0 | Some concerns | Undetected | No concerns | Major concerns | No concerns | Major concerns | Very low | ["Within-study bias", "Incoherence"] |

**Table S9** Evaluation of the Certainty of evidence of LH Using CINeMA Framework, mixed evidence.

| Comparison | Number of studies | Within-study bias | Reporting bias | Indirectness | Imprecision | Heterogeneity | Incoherence | Confidence rating | Reason(s) for downgrading |
| --- | --- | --- | --- | --- | --- | --- | --- | --- | --- |
| AA:AM | 1 | Some concerns | Undetected | No concerns | Some concerns | Some concerns | No concerns | moderate | ["Within-study bias", "Heterogeneity"] |
| AA:Control | 4 | Some concerns | Undetected | No concerns | Some concerns | Some concerns | No concerns | moderate | ["Within-study bias", "Heterogeneity"] |
| AM:Control | 5 | Some concerns | Undetected | No concerns | Some concerns | Some concerns | No concerns | moderate | ["Within-study bias", "Heterogeneity"] |
| AM:UM | 1 | Some concerns | Undetected | No concerns | Major concerns | No concerns | Major concerns | Very low | ["Within-study bias","Incoherence"] |
| AMandCM:Control | 1 | Some concerns | Undetected | No concerns | Some concerns | Some concerns | Major concerns | Very low | ["Within-study bias","Incoherence"] |
| AT:Control | 4 | Some concerns | Undetected | No concerns | Some concerns | Some concerns | Major concerns | Very low | ["Within-study bias","Incoherence"] |
| CA:EA | 1 | Some concerns | Undetected | No concerns | No concerns | Some concerns | Major concerns | Very low | ["Within-study bias","Incoherence"] |
| Control:EA | 4 | Some concerns | Undetected | No concerns | Some concerns | Some concerns | Major concerns | Very low | ["Within-study bias","Incoherence"] |
| Control:MB | 1 | Some concerns | Undetected | No concerns | Some concerns | Some concerns | Major concerns | Very low | ["Within-study bias","Incoherence"] |
| Control:SA | 1 | Some concerns | Undetected | No concerns | Major concerns | No concerns | Major concerns | Very low | ["Within-study bias","Incoherence"] |
| Control:WA | 4 | Some concerns | Undetected | No concerns | No concerns | Major concerns | Major concerns | Very low | ["Within-study bias","Incoherence"] |
| EA:HA | 2 | Some concerns | Undetected | No concerns | Major concerns | No concerns | Major concerns | Very low | ["Within-study bias","Incoherence"] |
| EA:MC | 1 | Some concerns | Undetected | No concerns | Major concerns | No concerns | Major concerns | Very low | ["Within-study bias","Incoherence"] |
| RA:SA | 1 | No concerns | Undetected | No concerns | Major concerns | No concerns | Major concerns | low | ["Incoherence"] |
| AA:AMandCM | 0 | Some concerns | Undetected | No concerns | Major concerns | No concerns | Major concerns | High | ["Within-study bias","Incoherence"] |
| AA:AT | 0 | Some concerns | Undetected | No concerns | Major concerns | No concerns | Major concerns | High | ["Within-study bias","Incoherence"] |
| AA:CA | 0 | Some concerns | Undetected | No concerns | Some concerns | Some concerns | Major concerns | High | ["Within-study bias","Incoherence"] |
| AA:EA | 0 | Some concerns | Undetected | No concerns | Some concerns | Some concerns | Major concerns | High | ["Within-study bias","Incoherence"] |
| AA:HA | 0 | Some concerns | Undetected | No concerns | Major concerns | No concerns | Major concerns | High | ["Within-study bias","Incoherence"] |
| AA:MB | 0 | Some concerns | Undetected | No concerns | Major concerns | No concerns | Major concerns | High | ["Within-study bias","Incoherence"] |
| AA:MC | 0 | Some concerns | Undetected | No concerns | Major concerns | No concerns | Major concerns | High | ["Within-study bias","Incoherence"] |
| AA:RA | 0 | Some concerns | Undetected | No concerns | Major concerns | No concerns | Major concerns | High | ["Within-study bias","Incoherence"] |
| AA:SA | 0 | Some concerns | Undetected | No concerns | Major concerns | No concerns | Major concerns | High | ["Within-study bias","Incoherence"] |
| AA:UM | 0 | Some concerns | Undetected | No concerns | Major concerns | No concerns | Major concerns | High | ["Within-study bias","Incoherence"] |
| AA:WA | 0 | Some concerns | Undetected | No concerns | Some concerns | Some concerns | Major concerns | High | ["Within-study bias","Incoherence"] |
| AM:AMandCM | 0 | Some concerns | Undetected | No concerns | Major concerns | No concerns | Major concerns | High | ["Within-study bias","Incoherence"] |
| AM:AT | 0 | Some concerns | Undetected | No concerns | Major concerns | No concerns | Major concerns | High | ["Within-study bias","Incoherence"] |
| AM:CA | 0 | Some concerns | Undetected | No concerns | Some concerns | Some concerns | Major concerns | High | ["Within-study bias","Incoherence"] |
| AM:EA | 0 | Some concerns | Undetected | No concerns | Some concerns | Some concerns | Major concerns | High | ["Within-study bias","Incoherence"] |
| AM:HA | 0 | Some concerns | Undetected | No concerns | Major concerns | No concerns | Major concerns | High | ["Within-study bias","Incoherence"] |
| AM:MB | 0 | Some concerns | Undetected | No concerns | Major concerns | No concerns | Major concerns | High | ["Within-study bias","Incoherence"] |
| AM:MC | 0 | Some concerns | Undetected | No concerns | Major concerns | No concerns | Major concerns | High | ["Within-study bias","Incoherence"] |
| AM:RA | 0 | Some concerns | Undetected | No concerns | Major concerns | No concerns | Major concerns | High | ["Within-study bias","Incoherence"] |
| AM:SA | 0 | Some concerns | Undetected | No concerns | Major concerns | No concerns | Major concerns | High | ["Within-study bias","Incoherence"] |
| AM:WA | 0 | Some concerns | Undetected | No concerns | Some concerns | Some concerns | Major concerns | High | ["Within-study bias","Incoherence"] |
| AMandCM:AT | 0 | Some concerns | Undetected | No concerns | Major concerns | No concerns | Major concerns | High | ["Within-study bias","Incoherence"] |
| AMandCM:CA | 0 | Some concerns | Undetected | No concerns | Major concerns | No concerns | Major concerns | High | ["Within-study bias","Incoherence"] |
| AMandCM:EA | 0 | Some concerns | Undetected | No concerns | Major concerns | No concerns | Major concerns | High | ["Within-study bias","Incoherence"] |
| AMandCM:HA | 0 | Some concerns | Undetected | No concerns | Major concerns | No concerns | Major concerns | High | ["Within-study bias","Incoherence"] |
| AMandCM:MB | 0 | Some concerns | Undetected | No concerns | Major concerns | No concerns | Major concerns | High | ["Within-study bias","Incoherence"] |
| AMandCM:MC | 0 | Some concerns | Undetected | No concerns | Major concerns | No concerns | Major concerns | High | ["Within-study bias","Incoherence"] |
| AMandCM:RA | 0 | Some concerns | Undetected | No concerns | Major concerns | No concerns | Major concerns | High | ["Within-study bias","Incoherence"] |
| AMandCM:SA | 0 | Some concerns | Undetected | No concerns | Major concerns | No concerns | Major concerns | High | ["Within-study bias","Incoherence"] |
| AMandCM:UM | 0 | Some concerns | Undetected | No concerns | Major concerns | No concerns | Major concerns | High | ["Within-study bias","Incoherence"] |
| AMandCM:WA | 0 | Some concerns | Undetected | No concerns | Major concerns | No concerns | Major concerns | High | ["Within-study bias","Incoherence"] |
| AT:CA | 0 | Some concerns | Undetected | No concerns | Some concerns | Some concerns | Major concerns | High | ["Within-study bias","Incoherence"] |
| AT:EA | 0 | Some concerns | Undetected | No concerns | Some concerns | Some concerns | Major concerns | High | ["Within-study bias","Incoherence"] |
| AT:HA | 0 | Some concerns | Undetected | No concerns | Major concerns | No concerns | Major concerns | High | ["Within-study bias","Incoherence"] |
| AT:MB | 0 | Some concerns | Undetected | No concerns | Major concerns | No concerns | Major concerns | High | ["Within-study bias","Incoherence"] |
| AT:MC | 0 | Some concerns | Undetected | No concerns | Major concerns | No concerns | Major concerns | High | ["Within-study bias","Incoherence"] |
| AT:RA | 0 | Some concerns | Undetected | No concerns | Major concerns | No concerns | Major concerns | High | ["Within-study bias","Incoherence"] |
| AT:SA | 0 | Some concerns | Undetected | No concerns | Major concerns | No concerns | Major concerns | High | ["Within-study bias","Incoherence"] |
| AT:UM | 0 | Some concerns | Undetected | No concerns | Major concerns | No concerns | Major concerns | High | ["Within-study bias","Incoherence"] |
| AT:WA | 0 | Some concerns | Undetected | No concerns | Some concerns | Some concerns | Major concerns | High | ["Within-study bias","Incoherence"] |
| CA:Control | 0 | Some concerns | Undetected | No concerns | No concerns | Some concerns | Major concerns | High | ["Within-study bias","Incoherence"] |
| CA:HA | 0 | Some concerns | Undetected | No concerns | Major concerns | No concerns | Major concerns | High | ["Within-study bias","Incoherence"] |
| CA:MB | 0 | Some concerns | Undetected | No concerns | Major concerns | No concerns | Major concerns | High | ["Within-study bias","Incoherence"] |
| CA:MC | 0 | Some concerns | Undetected | No concerns | Some concerns | Some concerns | Major concerns | High | ["Within-study bias","Incoherence"] |
| CA:RA | 0 | Some concerns | Undetected | No concerns | Major concerns | No concerns | Major concerns | High | ["Within-study bias","Incoherence"] |
| CA:SA | 0 | Some concerns | Undetected | No concerns | Some concerns | Some concerns | Major concerns | High | ["Within-study bias","Incoherence"] |
| CA:UM | 0 | Some concerns | Undetected | No concerns | Some concerns | Some concerns | Major concerns | High | ["Within-study bias","Incoherence"] |
| CA:WA | 0 | Some concerns | Undetected | No concerns | Major concerns | No concerns | Major concerns | High | ["Within-study bias","Incoherence"] |
| Control:HA | 0 | Some concerns | Undetected | No concerns | Major concerns | No concerns | Major concerns | High | ["Within-study bias","Incoherence"] |
| Control:MC | 0 | Some concerns | Undetected | No concerns | Major concerns | No concerns | Major concerns | High | ["Within-study bias","Incoherence"] |
| Control:RA | 0 | Some concerns | Undetected | No concerns | Major concerns | No concerns | Major concerns | High | ["Within-study bias","Incoherence"] |
| Control:UM | 0 | Some concerns | Undetected | No concerns | Major concerns | No concerns | Major concerns | High | ["Within-study bias","Incoherence"] |
| EA:MB | 0 | Some concerns | Undetected | No concerns | Major concerns | No concerns | Major concerns | High | ["Within-study bias","Incoherence"] |
| EA:RA | 0 | Some concerns | Undetected | No concerns | Major concerns | No concerns | Major concerns | High | ["Within-study bias","Incoherence"] |
| EA:SA | 0 | Some concerns | Undetected | No concerns | Major concerns | No concerns | Major concerns | High | ["Within-study bias","Incoherence"] |
| EA:UM | 0 | Some concerns | Undetected | No concerns | Major concerns | No concerns | Major concerns | High | ["Within-study bias","Incoherence"] |
| EA:WA | 0 | Some concerns | Undetected | No concerns | Some concerns | Some concerns | Major concerns | High | ["Within-study bias","Incoherence"] |
| HA:MB | 0 | Some concerns | Undetected | No concerns | Major concerns | No concerns | Major concerns | High | ["Within-study bias","Incoherence"] |
| HA:MC | 0 | Some concerns | Undetected | No concerns | Major concerns | No concerns | Major concerns | High | ["Within-study bias","Incoherence"] |
| HA:RA | 0 | Some concerns | Undetected | No concerns | Major concerns | No concerns | Major concerns | High | ["Within-study bias","Incoherence"] |
| HA:SA | 0 | Some concerns | Undetected | No concerns | Major concerns | No concerns | Major concerns | High | ["Within-study bias","Incoherence"] |
| HA:UM | 0 | Some concerns | Undetected | No concerns | Major concerns | No concerns | Major concerns | High | ["Within-study bias","Incoherence"] |
| HA:WA | 0 | Some concerns | Undetected | No concerns | Major concerns | No concerns | Major concerns | High | ["Within-study bias","Incoherence"] |
| MB:MC | 0 | Some concerns | Undetected | No concerns | Major concerns | No concerns | Major concerns | High | ["Within-study bias","Incoherence"] |
| MB:RA | 0 | Some concerns | Undetected | No concerns | Major concerns | No concerns | Major concerns | High | ["Within-study bias","Incoherence"] |
| MB:SA | 0 | Some concerns | Undetected | No concerns | Major concerns | No concerns | Major concerns | High | ["Within-study bias","Incoherence"] |
| MB:UM | 0 | Some concerns | Undetected | No concerns | Major concerns | No concerns | Major concerns | High | ["Within-study bias","Incoherence"] |
| MB:WA | 0 | Some concerns | Undetected | No concerns | Major concerns | No concerns | Major concerns | High | ["Within-study bias","Incoherence"] |
| MC:RA | 0 | Some concerns | Undetected | No concerns | Major concerns | No concerns | Major concerns | High | ["Within-study bias","Incoherence"] |
| MC:SA | 0 | Some concerns | Undetected | No concerns | Major concerns | No concerns | Major concerns | High | ["Within-study bias","Incoherence"] |
| MC:UM | 0 | Some concerns | Undetected | No concerns | Major concerns | No concerns | Major concerns | High | ["Within-study bias","Incoherence"] |
| MC:WA | 0 | Some concerns | Undetected | No concerns | Major concerns | No concerns | Major concerns | High | ["Within-study bias","Incoherence"] |
| RA:UM | 0 | Some concerns | Undetected | No concerns | Major concerns | No concerns | Major concerns | High | ["Within-study bias","Incoherence"] |
| RA:WA | 0 | Some concerns | Undetected | No concerns | Major concerns | No concerns | Major concerns | High | ["Within-study bias","Incoherence"] |
| SA:UM | 0 | Some concerns | Undetected | No concerns | Major concerns | No concerns | Major concerns | High | ["Within-study bias","Incoherence"] |
| SA:WA | 0 | Some concerns | Undetected | No concerns | Major concerns | No concerns | Major concerns | High | ["Within-study bias","Incoherence"] |
| UM:WA | 0 | Some concerns | Undetected | No concerns | Major concerns | No concerns | Major concerns | High | ["Within-study bias","Incoherence"] |

**Figure S1** Kupperman score local inconsistency test

**Figure S2** FSH local inconsistency test

**Figure S3** LH local inconsistency test

**Figure S4** E2 local inconsistency test

**Figure S5** Kupperman score funnel chart.

**Figure S6** FSH funnel chart.

**Figure S7** LH funnel chart.

**Figure S8** E2 funnel chart.

**Figure S9** Subgroup analysis for E2.

Covarate 1 represents treatment duration less than 30 minutes, and 2 represents treatment duration greater than or equal to 30 minutes.

**Figure S10** Subgroup analysis for Kupperman Scroe.

**Figure S11** Subgroup analysis for FSH.

**Figure S12** Subgroup analysis for LH.

**Figure S13** SUCRA ranking of various interventions in different outcomes under a complete network.

Kuperman, FSH, and LH are sorted as low values, while E2 is sorted as high values. Points and values represent the percentage of SUCRA.

**Figure S14** SUCRA changes after deleting a single intervention node.

The horizontal axis represents the deleted treatment, and the vertical axis represents the evaluated treatment; Red indicates an increase in SUCRA, blue indicates a decrease in SUCRA, in percentage points. The white diagonal line indicates that the deleted treatment is no longer involved in the left model.

**Figure S15** Removing the impact of each treatment node on the overall treatment hierarchy.

The horizontal axis represents the largest absolute SUCRA change among all retained treatments; The orange dots indicate that the optimal treatment has changed after deleting the node.

**Figure S16** Evidence network for each outcome.

The node size is proportional to the cumulative sample size of the treatment, and the line thickness is proportional to the number of direct comparative studies.
